# StkP- and PhpP-Mediated Posttranslational Modifications Modulate the S. pneumoniae Metabolism, Polysaccharide Capsule, and Virulence

**DOI:** 10.1128/iai.00296-22

**Published:** 2023-03-06

**Authors:** Sashi Kant, Youcheng Sun, Vijay Pancholi

**Affiliations:** a Department of Pathology, Ohio State University College of Medicine, Columbus, Ohio, USA; University of Illinois at Chicago

**Keywords:** *Streptococcus pneumoniae*, virulence, Ser/Thr phosphatase (PhpP), Ser/Thr kinase (StkP), CcpA, capsule, posttranslational modification, transcriptional regulation, global-gene transcriptome, metabolism, polysaccharide capsule, transcription factor, pathogenesis, catabolite control protein (CcpA)

## Abstract

Pneumococcal Ser/Thr kinase (StkP) and its cognate phosphatase (PhpP) play a crucial role in bacterial cytokinesis. However, their individual and reciprocal metabolic and virulence regulation-related functions have yet to be adequately investigated in encapsulated pneumococci. Here, we demonstrate that the encapsulated pneumococcal strain D39-derived D39ΔPhpP and D39ΔStkP mutants displayed differential cell division defects and growth patterns when grown in chemically defined media supplemented with glucose or nonglucose sugars as the sole carbon source. Microscopic and biochemical analyses supported by RNA-seq-based global transcriptomic analyses of these mutants revealed significantly down- and upregulated polysaccharide capsule formation and *cps2* genes in D39ΔPhpP and D39ΔStkP mutants, respectively. While StkP and PhpP individually regulated several unique genes, they also participated in sharing the regulation of the same set of differentially regulated genes. C*ps2* genes were reciprocally regulated in part by the StkP/PhpP-mediated reversible phosphorylation but independent of the MapZ-regulated cell division process. StkP-mediated dose-dependent phosphorylation of CcpA proportionately inhibited CcpA-binding to P*cps2A*, supporting increased *cps2* gene expression and capsule formation in D39ΔStkP. While the attenuation of the D39ΔPhpP mutant in two mouse infection models corroborated with several downregulated capsules-, virulence-, and phosphotransferase systems (PTS)-related genes, the D39ΔStkP mutant with increased amounts of polysaccharide capsules displayed significantly decreased virulence in mice compared to the D39 wild-type, but more virulence compared to D39ΔPhpP. NanoString technology-based inflammation-related gene expression and Meso Scale Discovery-based multiplex chemokine analysis of human lung cells cocultured with these mutants confirmed their distinct virulence phenotypes. StkP and PhpP may, therefore, serve as critical therapeutic targets.

## INTRODUCTION

Streptococcus pneumoniae (pneumococcus), a Gram-positive pathogen, causes various invasive diseases, including pneumonia, meningitis, septicemia, and otitis media (1–3). According to the World Health Organization (WHO) estimate, pneumococcal infections result in the deaths of more than 1.6 million people, including more than 800,000 children, every year worldwide despite the availability of multivalent capsule-based vaccines (4, 5). The polysaccharide capsule plays a decisive role in almost all stages of the pneumococcal life cycle, from early colonization, phagocytic evasion, and proliferation to reemergence (6). The most remarkable characteristic of more than 90 serotypes of pneumococcal caps is genetic plasticity, which translates into a shift in capsular types (7). The efficacy of the current vaccine limitation is attributed to the emergence and switching of serotypes (8, 9). Hence, an alternative noncapsule surface virulence factor(s) (protein)-based vaccine approach has gained importance (10, 11). Nevertheless, among several pneumococcal virulence factors (6, 12), the polysaccharide capsule (CPS) is still recognized as the major virulence factor (13–15), and pneumococcal colonization is the critical determining step in pneumococcal diseases (6, 14, 16).

Eukaryote-type transmembrane serine/threonine kinases (STK) containing a signature penicillin-binding protein (PBP) and Ser/Thr kinase-associated (PASTA) domain, and their cognate phosphatases (STPs) in Gram-positive pathogens, including S. pneumoniae, have lately received substantial attention due to their ability to regulate several cellular functions. These include metabolic fitness, cell division, cell wall biosynthesis, spore formation, antimicrobial resistance, and virulence (17, 18). Many of these functions are indirectly regulated through the modulation of the functional status of two-component system (TCS) regulators involved in the direct transcriptional regulation of genes responsible for such functions (19). Homologs of STK and STP enzymes in S. pneumoniae are identified as StkP and PhpP (20). After initial recognition of the role of StkP in S. pneumoniae competence and stress resistance (20–22), StkP and PhpP were most importantly implicated in their role in pneumococcus cytokinesis and morphogenesis (23–25). This role, however, is seen as an outcome of posttranslational modifications via reversible phosphorylation of critical Thr-residues of proteins involved in translation, cell division and cell wall peptidoglycan synthesis, lipid metabolism, and other cellular activities, including transcription, carbohydrate metabolism, energy metabolism, and DNA repair, replication and modification (26, 27).

Among all these StkP/PhpP-mediated activities, the primary focus has been modulating the function of several cell division proteins (28), such as DivIVA (25, 29), LocZ/MapZ (30, 31), MacP (32), EloR (33), PBP2X (34), FtsZ (23), and GpsB (31) and cell wall peptidoglycan synthesis proteins (Glms and GpsA) (26, 27). In addition, StkP and PhpP have been incriminated in reversible phosphorylation of a pneumococcal orphan transcriptional regulator, RitR, and a two-component regulator RR06 (35–37). Although the functions of StkP and PhpP are extensively studied to understand their roles in pneumococcal cell division and morphology (25, 28, 38, 39), all these studies, including the recent studies on PhpP and StkP (26, 27, 40, 41), are carried out using nonencapsulated pneumococcal strains. While not explicitly clear, such a trend has likely persisted with the presumption that polysaccharide capsules might interfere with the cell division process. The observed growth defects resulting from defective cell division in the absence of StkP and PhpP and the ability of StkP to target proteins of many cellular activities besides cell division (26, 27) may directly or indirectly contribute to pneumococcal virulence. However, no systematic reports are available to understand the role of StkP/PhpP in the metabolism, virulence, and pathogenesis of encapsulated pneumococci. Earlier, we reported strain-specific phenotypic characteristics of the encapsulated pneumococcus PhpP knockout mutant derived from the encapsulated S. pneumoniae D39 and 6A strains (36). However, these results did not conclusively resolve the role of PhpP in pneumococcal virulence, especially in comparison to the isogenic StkP mutant. Ser/Thr phosphatases (homologs of PhpP) in many other Gram-positive pathogens, including S. pyogenes, *S.aureus*, and S. agalactiae, have been reported to play an essential role in bacterial virulence (42–44).

Although noncapsular surface-associated and secreted proteins also play a substantial role in pneumococcal virulence (10, 11), the pneumococcal polysaccharide capsule is still considered the major virulence factor. Hence, all current pneumococcal immunization efforts focus on producing protective antibodies against various polysaccharide capsules of prevalent serotypes (45, 46). It is well-established that the amount of capsular polysaccharide present on the surface is directly related to the transcript levels of the *cps* locus (*spd_0315*- *spd_0333*), which is located between *dexB* and *aliA g*enes in S. pneumoniae D39 (47). The capsule production is also directly related to the pneumococcal carriage across several serotypes (48, 49). The *cps* promoter (P*cps*) regulates the transcription of *cps* genes. Predicted *in silico* bioinformatic analysis (50), direct *Pcps*-binding studies (51), and recent findings on VncR (52) have shown that several candidate regulators control transcription of the *cps* locus genes. These include CcpA, a GntR family transcriptional regulator SPD_0064, a MarR family transcriptional regulator SPD_0379, DNA-binding protein HU, CodY, GlnR, and RitR (51). Although CcpA ensures optimal metabolic fitness in S. pneumoniae (53), its role in modulating capsule production, metabolic fitness, and ultimately virulence of S. pneumoniae through StkP activity is presently unknown.

In the present investigation, we have created and characterized the type-2 encapsulated pneumococcus D39 strain-derived mutants lacking StkP or PhpP. We have demonstrated that StkP and PhpP-mediated reversible phosphorylation reciprocally regulate genes encoding the entire capsule operon and corresponding expression of the capsule. Further, this regulation is mediated via the reciprocal StkP/PhpP-mediated reversible phosphorylation status of catabolite control protein (CcpA), resulting in differential regulation of the transport and metabolism of nonglucose sugars. The latter plays a vital role in forming building block sugar components of the pneumococcal polysaccharide capsule and critical sugars required for colonization and infection (53–56). Biochemical, proteomic, and global transcriptome analyses of D39ΔStkP and D39ΔPhpP mutants have revealed that StkP and PhpP independently, together, and reciprocally modulate the expression of several bacterial genes. Further, NanoString- and Meso Scale Discovery Electrochemiluminescence (MSD)-based inflammatory genes transcriptome analyses and multiplex cytokine/chemokine analyses of human lung cells cocultured with the pneumococcus mutants establish that StkP and PhpP modulate host inflammatory responses, and thus together, they play a pivotal role in pneumococcus virulence and pathogenesis.

## RESULTS

### Phenotypic characteristics of D39ΔPhpP and D39ΔStkP mutants and their corresponding complemented strains.

**FIG 1:**
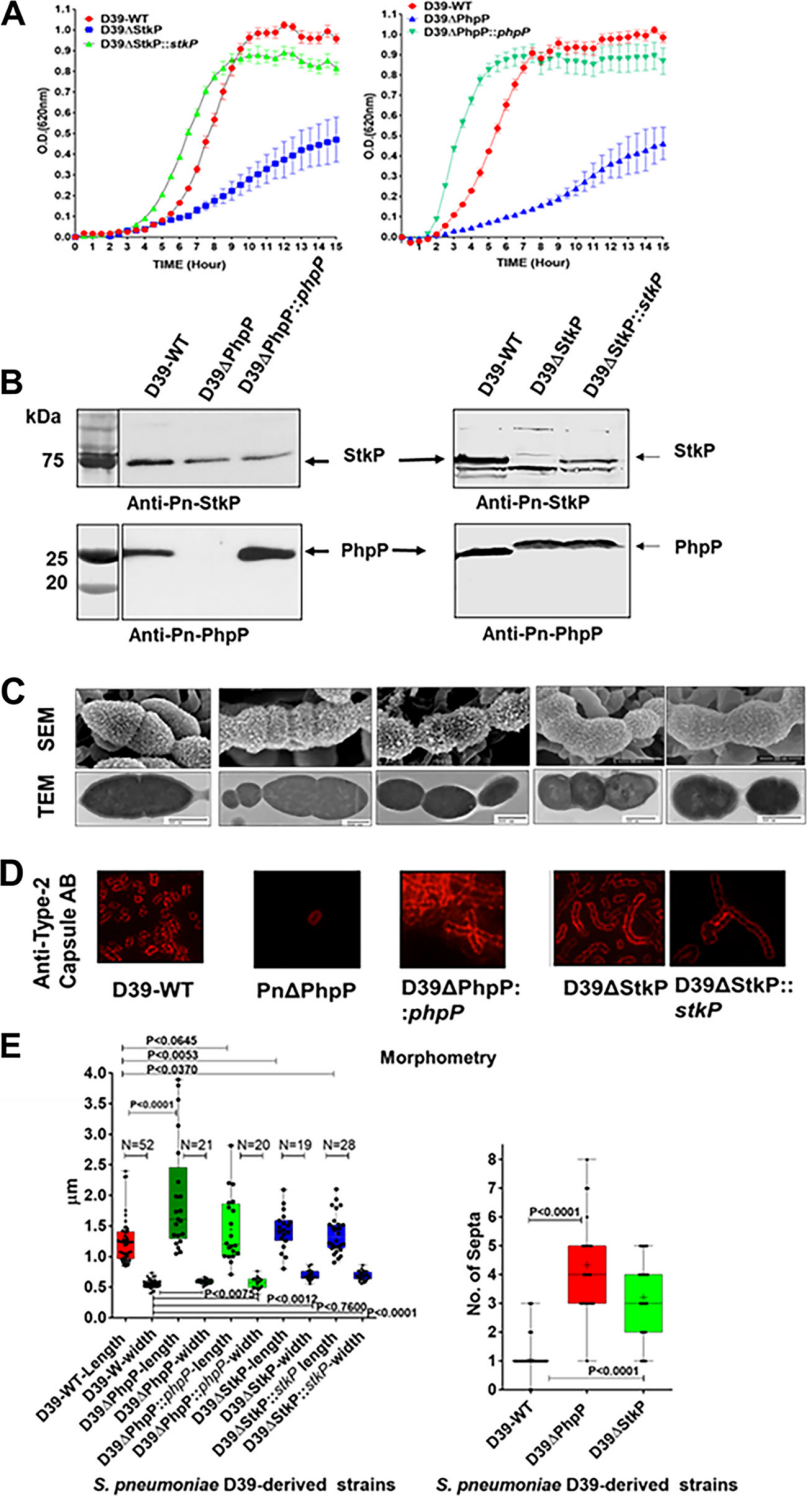
Characterization of isogenic D39 S. pneumoniae-derived ΔStkP and ΔPhpP mutants and complemented strains, and modulation of the polysaccharide capsule formation. (A) Growth curves of the D39-WT, D39ΔStkP, D39ΔPhpP, and *stkP*- and *phpP*-complemented mutant strains grown in C+Y (1% wt/vol glucose) medium for 15 h, as described in Materials and Methods. Error bar indicates average reading ± SD obtained from three independent cultures. (B) Western immunoblot analysis shows the presence and absence of PhpP and StkP in the S. pneumoniae D39 wild-type (D39-WT), mutants lacking PhpP or StkP (D39ΔPhpP, D39ΔStkP), and the mutants complemented with the wild-type *phpP/stkP* gene (D39ΔPhpP::*phpP*, D39ΔStkP::*stkP*) using affinity-purified rabbit polyclonal anti-PhpP and Ant-StkP antibodies. The numbers on the left depict the positions of the molecular weight markers. (C) Scanning electron microscopy (SEM, upper panel); transmission electron microscopy (TEM, middle panel); and (D) immunofluorescence microscopy (IF, lower panel) of S. pneumoniae D39 strains as indicated. Scale bar = 500 nm for each panel of SEM and TEM for the respective mutant strain as shown. (D) Immunofluorescence microscopy was performed using type-2 specific antibody preadsorbed with heat-killed S. pneumoniae nonencapsulated D39-derived R6 strain and corresponding Cy3-labeled antirabbit IgG conjugated antibody. (E) Quantitative morphometric analysis determining lengths, widths, and number of septa present in different pneumococcal strains. N = number of fields, each representing an average of randomly selected 4 to 6 bacteria. Statistical analysis as indicated in the text. *P* value <0.05 was treated as a significant difference.

In the present study, we used the markerless D39ΔPhpP mutant, as previously reported (36). D39ΔStkP was created with *aad9* (spectinomycin resistance) as the differential resistance marker. Genome sequence analysis of the D39ΔStkP mutant versus D39 wild type did not show any changes in the sequence except the absence of *stkP*. On the other hand, the D39ΔPhpP mutant grown in the THY medium showed the deletion of one nucleotide, G, between residues number 1995571 and 199572 (in the 4th passage of the mutant but not in the 1st passage). This mutation was found to be localized in the *cbpA/spd_2017* gene spanning between nucleotides 1995045 and 1997150, translating into truncation of the last 56 amino acids and a loss of the last repeat region of the CbpA protein (Fig. S1). Both mutants grew slower in C+Y chemically defined medium (CDM). The wild-type gene complemented D39ΔStkP and D39ΔPhpP mutant strains regained growth patterns akin to the wild-type strain (Fig. 1A). Western blot analysis of the whole-cell lysates of D39ΔPhpP and D39ΔStkP mutants revealed the absence of the expression of PhpP and StkP and the corresponding presence of StkP and PhpP, respectively (Fig. 1B). In a similar assay, the *phpP-* and *stkP*-complemented mutant strains (D39ΔPhpP::*phpP* and D39ΔStkP::*stkP*) showed the restoration of the expression of the corresponding encoded proteins confirming the mutant integrity (Fig. 1B). These mutants and complemented strains were then subjected to various biological and biochemical assays to understand the contribution of the PhpP and StkP proteins to different metabolic, regulatory, and virulence properties of the encapsulated strain of S. pneumoniae D39, as described in subsequent sections.

Phenotypic characteristics of pneumococcal strains lacking StkP have been extensively studied (28, 31). Characteristic features of D39ΔPhpP mutants were elongated shapes with significant variations and irregularity with multiple parallel septa resulting in a long-chain formation. Similarly, the D39ΔStkP mutant strain was also found to be defective cell division, showing irregular septum with the appearance of ovoid morphotypes as revealed by scanning and transmission electron microscopy (Fig. 1C and E). The length and width sizes of D39 mutants were significantly larger (*P* < 0.01) compared to the D39 wild-type strain. The morphometric analysis of complemented strains showed an overall trend of canonical shape and size of the wild-type strain (Fig. 1E). However, in this first report on PhpP and StkP mutants derived from the parent encapsulated strain D39, we were able to demonstrate additional essential features. As shown in Fig. 1C, transmission and scanning electron microscopy (TEM and SEM) of the wild-type D39 strain showed several distinct, irregular appendages signifying the surface-decorated capsule. However, the cell surface of D39ΔPhpP showed a relatively much smoother surface, indicating the reduced formation of the capsular polysaccharide. The *phpP*-complemented strain demonstrated the restoration of this lost function and mimicked the wild-type cell surface structure. The D39ΔStkP mutant, on the other hand, showed notably increased amounts of the capsule, suggesting the role of StkP/PhpP in reciprocal regulation of the capsule formation. (Fig. 1C). The differential putative capsule formation feature was further examined by immunofluorescence microscopy using an antipolysaccharide type-2 antibody that was preadsorbed with heat-killed D39-derived nonencapsulated R6 strain. The increased and decreased antibody binding to the D39ΔStkP and D39ΔPhpP mutant strains, respecticely, (Fig. 1D) strongly indicated that pneumococcal StkP and PhpP have a role in the modulation of capsule production.

### PhpP and StkP reciprocally regulate capsule formation in S. pneumoniae.

**FIG 2:**
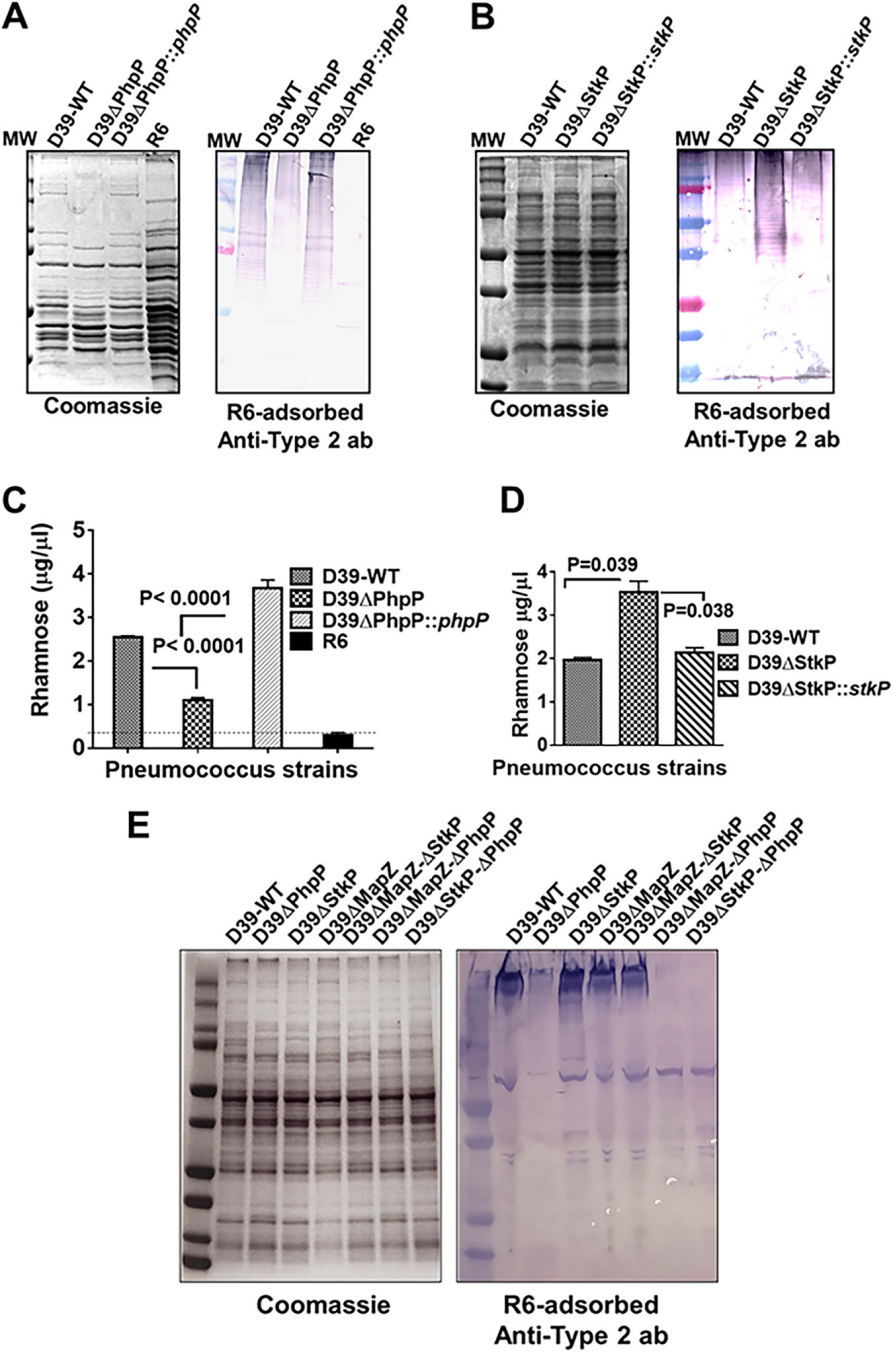
Polysaccharide capsule estimation in S. pneumoniae D39 strain-derived ΔPhpP and ΔStkP mutants and their corresponding complemented strains. Qualitative Western immunoblot analysis shows decreased and increased capsule formation in (A) D39ΔPhpP and (B) D39ΔStkP mutants, respectively, using type-2 polysaccharide capsule-specific antibody as described above for IF microscopy. The respective complemented strains show the wild-type capsule formation in each. Quantitative estimation of rhamnose contents in the polysaccharide capsule extracted from the (C) D39ΔPhpP mutant and (D) D39ΔStkP mutant strains and their comparison to the parent wild-type strain and their corresponding complemented strains. Each error bar represents an average (± SD) quantity of rhamnose detected in the polysaccharide extracted from three individually grown cultures. *P* < 0.05 was treated as a significant difference and was calculated using GraphPad Prism 6. S. pneumoniae R6 strain (nonencapsulated) was used as a negative control. (E) The left panel is Coomassie-stained SDS-PAGE gel and serves as a loading control for the duplicate right panel representing Western immunoblot-based qualitative analysis of polysaccharide capsule in D39-derived single ΔStkP, ΔPhpP, ΔMapZ, and ΔPhpP mutants and ΔMapZ-ΔStkP, ΔMapZ-ΔPhpP, and ΔStkP-ΔPhpP double mutants, using type-2 polysaccharide capsule-specific antibody as described above for IF microscopy.

We estimated the amount of polysaccharide capsules extracted from an equal optical density (OD) volume-equilibrated pneumococcal culture suspensions to confirm the microscopic findings. Qualitative analysis of the polysaccharide capsule in Western immunoblots assays using the anti-type-2 capsule antibody revealed significantly decreased capsule-specific reactivity in the D39ΔPhpP mutant relative to the wild-type D39 strain and the *phpP*-complemented ΔPhpP strain (Fig. 2A). The D39ΔStkP mutant, on the other hand, showed a substantially increased amount of polysaccharide capsule, which was restored in the *stkP*-complemented ΔStkP mutant to the wild-type level (Fig. 2B).

We further substantiated these findings by examining rhamnose-sugar contents quantitatively in each capsule extract (Fig. 2C and D). Compared to R6 (negative capsule derivative of D39 strain and negative control), the wild-type strain showed the presence of rhamnose content in the range of 2 to 2.5 μg/μL. Furthermore, the results obtained from the mutants showed significantly decreased (*P* < 0.0001) and increased (*P* = 0.039) rhamnose contents in the capsule extracted from D39ΔPhpP (Fig. 2C) and D39ΔStkP (Fig. 2D) mutants, respectively, compared with the wild-type D39 and their respective complemented strains. Together, these data indicated that the StkP- and PhpP-mediated reversible phosphorylation contributed to decreasing and increasing capsule production in pneumococci through a specific mechanism.

### StkP/PhpP-modulated capsule formation is independent of the MapZ-regulated cell division in S. pneumoniae.

MapZ or LocZ protein, as a crucial cell division protein, is involved in proper septum placement in S. pneumoniae (30, 31) by regulating its binding to peptidoglycan and other accessory proteins (57, 58). Similarly, peptidoglycan and capsule biosynthesis are coordinately regulated in Gram-positive bacteria (59). StkP/PhpP-mediated reversible phosphorylation of MapZ plays a specific role in the recruitment of FtsZ at the septum (25, 31, 58). This event initiates the assembly of the other division proteins at the septum of nonencapsulated pneumococcus (31). Hence, we investigated whether the MapZ-regulated cell division process indirectly influenced the altered capsule formation in the D39ΔPhpP and D39ΔStkP mutant in the encapsulated D39 pneumococcus strain. To address this, we created a D39ΔMapZ mutant strain by replacing the *mapZ* gene with the promoterless *kanR* gene employing the allelic replacement method. The genetically confirmed D39ΔMapZ mutant showed expected cell division defects (Fig. S2), as previously demonstrated in the nonencapsulated pneumococcus strain (28–30). However, this mutant with intact StkP and PhpP did not reveal any difference in the capsule formation. We, therefore, surmised that the absence of the peptidoglycan-associated MapZ did not influence differential capsule formation by cell wall-associated StkP and cytoplasmic PhpP and, thus, did not interfere in the capsule biosynthesis in pneumococcus.

Since StkP and PhpP are also involved in cell division, we investigated further to determine to what extent MapZ influences the modulatory activity of StkP and PhpP in capsule formation. To that end, we created double mutants, D39ΔMapZ-ΔStkP (*kan^R^*, *aad9^R^*), D39ΔMapZ-ΔPhpP (*kan^R^*), and D39ΔStkP-ΔPhpP (*aad9^R^*), as described in the Materials and Methods. All mutants, including D39ΔStkP-ΔPhpP, showed slow growth (maximum OD 0.3 to 0.4) associated with cell division defects in the initial passage. However, the growth patterns of all mutants except D39ΔStkP-ΔPhpP improved (OD 0.8 to 0.9) when the starting inoculum for each of these strains was used from the consecutively multiple-passaged culture, as described in the later section. Recently, similar observations have been reported (27, 40) for StkP/PhpP mutants explaining that multiple passages of StkP and PhpP mutants accumulate suppressor mutants, which do not influence cell division phenotype but are responsible for enhanced growth. Our limited genome analysis of the D39ΔPhpP mutant after four passages concurs with this notion. Most mutants showed loss of chains and wild-type ovo-coccoid forms and predominance of diverse sizes of aggregated forms of round or deformed shape of coccoid structures resulting from the unevenly arranged septa formation. (Fig. S2D).

To determine the modulation of polysaccharide capsule formation in these mutants, whole-cell lysates obtained from each mutant at their normalized total protein concentration (1 mg/mL) were subjected to Western blot analysis using the anti-type-2 polysaccharide antibody. The result showed a significant decrease in capsule formation in the mutant lacking PhpP. Thus, D39ΔPhpP, D39ΔMapZ-PhpP, and D39ΔStkP-PhpP showed negligible amounts or the absence of polysaccharide capsules. At the same time, capsule contents in mutants lacking StkP and/or MapZ were more than or equal to the wild-type D39 strain, confirming that the polysaccharide capsule formation is independent of MapZ-associated cell division defect (Fig. 2E, Fig. S2C).

### PhpP and StkP differentially influence the expression of a variety of genes and reciprocally regulate the transcription of capsule-related genes.

**FIG 3:**
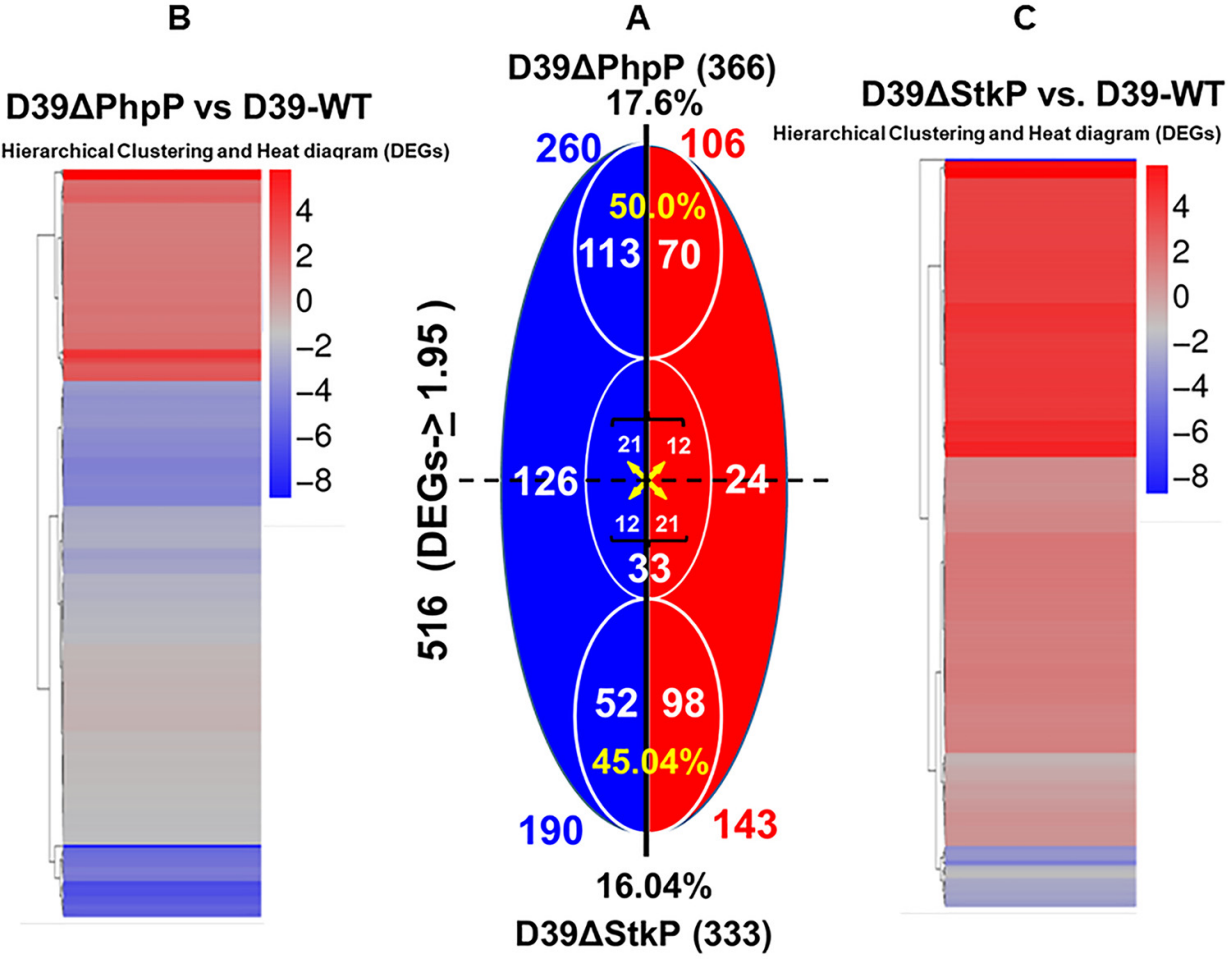
Summary of differentially regulated genes in D39ΔPhpP and D39ΔStkP mutants in comparison to the wild-type D39 S. pneumoniae D39 strain. RNA-seq-based differential gene expression analysis of D39ΔPhpP versus D39-WT and D39ΔStkP versus D39-WT based on the average of three independent biological replicates (GEO database no. GSE113337). (A) Venn diagram shows the number of differential genes in *S. pneumonia* D39-WT-derived mutant strains. The upper half of the diagram represents the distribution of DEGs in D39ΔPhpP, and the lower half represents D39ΔStkP. Blue and red quadrants represent down- and upregulated genes, respectively. The upper and lower circles represent numbers of DEGs found exclusively in D39ΔPhpP and D39ΔStkP mutants. The middle circle represents the number of DEGs reciprocally regulated in these mutants. The numbers at the dotted line represent DEGs either upregulated or downregulated in both mutants. Overall gene expression analysis of (B) D39ΔPhpP versus D39-WT and (C) D39ΔStkP versus D39-WT as depicted by heatmap hierarchical clustering of genes. Numbers within the diagram indicate the number of DEGs. The heatmap scale (± Log_2_) is shown on the right upper side of panels B and C.

**FIG 4:**
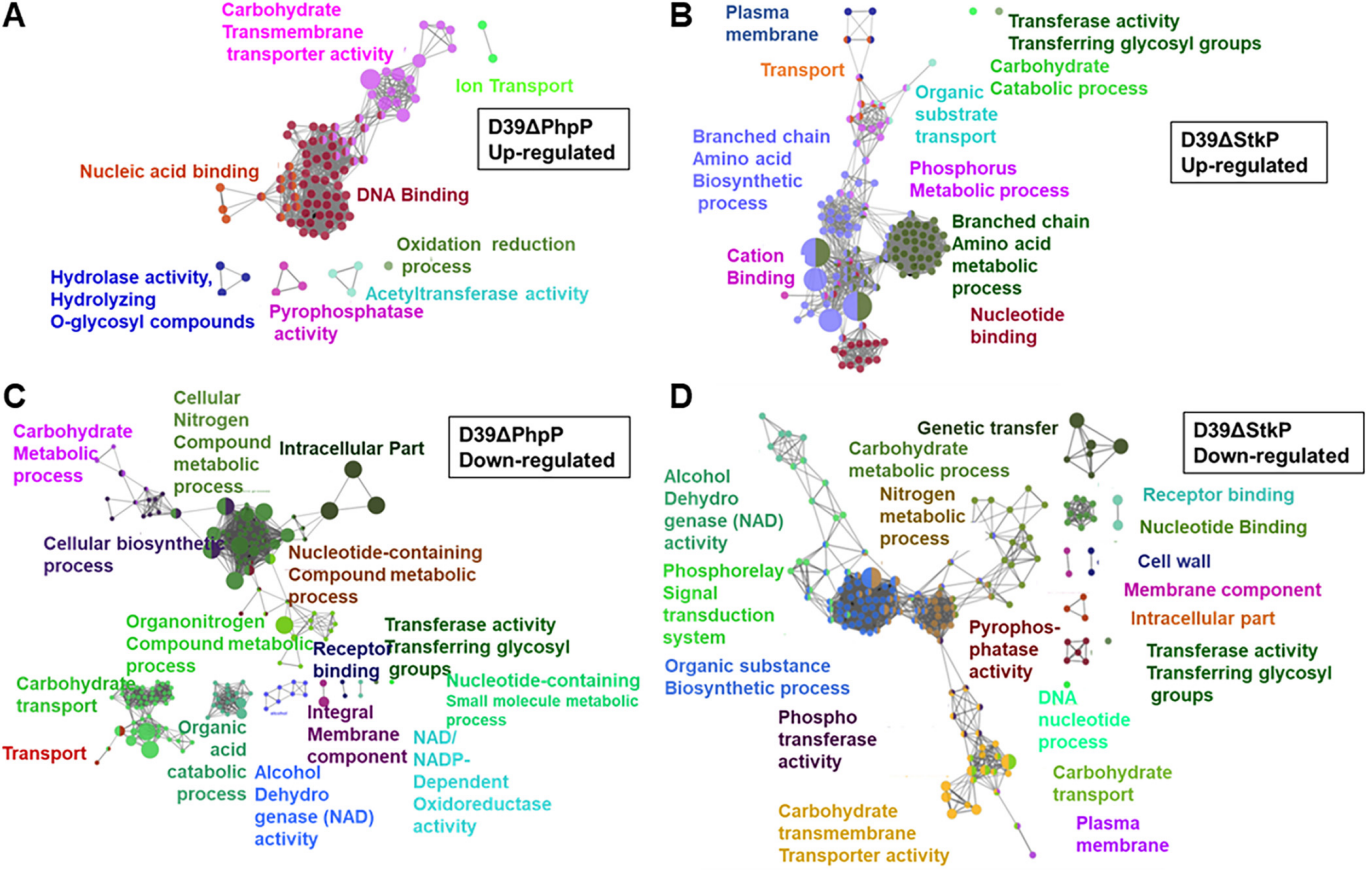
ClueGo- and Cytoscape-based analysis of the clustered DEGs in D39ΔPhpP and D39ΔStkP. (A and B) upregulated and (C and D) downregulated clustered DEG networks in D39ΔPhpP and D39ΔStkP mutants. The analysis was performed as described in Materials and Methods.

Since the relative activity of these enzymes changes based on the intracellular metabolic environment and cell wall stress (27, 60, 61), the primary focus of this study was to dissect the molecular and cellular bases of regulatory functions of PhpP/StkP in encapsulated pneumococcus besides its role in cytokinesis. Thus, to better understand the impact of StkP/PhpP-mediated reversible phosphorylation in the transcriptional regulation of genes in pneumococcus, we first examined global mRNA expression levels of genes in D39 wild-type, and isogenic D39ΔPhpP and D39ΔStkP mutant strains employing Illumina-based RNA-seq transcriptome analysis. This analysis was conducted using high-quality DNA-free total RNA extracted from cultures grown to the mid-log-phase. The data of cDNA libraries of an average of 160 bp inserts and their paired sequence data derived from nine samples (three samples each of the wild type and two mutants) were then analyzed and deposited in the GEO database (GEO accession no. GSE113337). At least 1.5 million reads with more than 90% of the entire genome were resolved for each sample using SOAP aligner/SOAP2 alignment software tools (62) and BOWTIE (63). Based on our previously used statistical analysis tools (64), we were able to obtain a robust analysis of all data with SOAP2 tools, which detected transcripts of more than 98% of genes out of a total of 2,076 genes of the D39 genome (NC_008533.1/CP000410.1) (65). The stringent criteria employed (≥1.95-fold) in the present study (66) enabled us to identify with high confidence a total of 516 differentially expressed genes (DEGs) (25.6% of total 2,076 genes) in D39ΔPhpP (366 genes) (Fig. 3A and B) and D39ΔStkP (333 genes) mutant strains (Fig. 3A and C, Table S1). Among these DEGs, a group of 183 (50%) and 150 DEGs (45.04%) were unique to D39ΔPhpP (Table S2A) and D39ΔStkP mutants (Table S2B), respectively. PhpP and StkP independently regulated these genes. Both mutants displayed similar patterns of differential regulations for 150 genes (24 upregulated and 126 downregulated genes). (Table S3). The reciprocal regulation was observed for only 33 genes (6.39% of the total DEGs in these mutants) (Table 1). In addition to the *phpP* gene (SPD_1543), the genome of S. pneumoniae D39 also consists of two additional genes encoding the metal-binding Ser/Thr phosphatase family, SPD_0539 (834 bp/277 aa) and SPD_1061 (729 bp/242 aa), which harbor conserved catalytic residues reported in PhpP. Irrespective of their sparse protein sequence identities and similarities with PhpP, the expression levels of these genes were found to be unaltered (1.15- and 1.2-fold increased transcript abundance). Further, the expression level of immediate upstream and downstream flanking genes of *phpP* and *stkP* genes also remained unaltered. Hence, the observed changes in D39ΔPhpP and D39ΔStkP are effectively due to the deletion of the *phpP* or *stkP* (*spd_1543/1542*) gene. The overall gene expression profiles supported by ClueGo analysis (Fig. 4) in these two mutants indicated that both PhpP and StkP participated independently, reciprocally, and together in regulating several carbohydrate transport-metabolism-, nucleotide-purine/pyridine metabolism-, protein synthesis-, and iron transport-related genes. This differential global gene expression repertoire resulted in the observed phenotypes of S. pneumoniae described above (Figs. 1 and 2).

**TABLE 1:** Reciprocal-capsule and noncapsular genes in D39ΔPhpP and D39ΔStkP mutants

			D39DPhpP vs D39WT		D39DStkP vs D39WT	
Gene ID	Gene name		Fold change^*a*^	q value	Fold change^*a*^	q value
Capsule-encoding genes						
**SPD_0315**	Lytr family transcriptional regulator (Cps2A)		−**14.7**	0	**1.50**	7.06E-29
**SPD_0316**	Hypothetical protein (Cps2B)		−**18.2**	5.06E-127	1.46	1.09E-19
**SPD_0317**	Capsular polysaccharide biosynthesis protein Cps2C		−**17.6**	0	1.46	1.48E-14
**SPD_0318**	Tyrosine protein kinase (Cps2D)		−**17.1**	0	1.37	2.76E-11
**SPD_0319**	Galactosyl transferase (Cps2E)		−**15.1**	5.81E-113	**1.56**	4.81E-31
**SPD_0320**	Glycosyl transferase (Cps2T)		−**13.8**	0	**1.71**	7.69E-37
**SPD_0321**	Glycosyl transferase (Cps2F)		−**12.9**	0	**2.01**	4.74E-58
**SPD_0322**	Glycoside hydrolase (Cps2G)		−**11.8**	1.76E-87	**2.14**	8.59E-79
**SPD_0323**	Membrane protein (Cps2H)		−**9.48**	0	**2.0**	2.98E-37
**SPD_0324**	Glycosyl transferase family 1 (Cps2I)		−**8.88**	2.86E-245	**2.07**	2.32E-48
**SPD_0325**	Hypothetical protein (Cps2J)		−**8.89**	1.38E-225	**1.95**	6.64E-39
**SPD_0326**	UDP-glucose dehydrogenase (Cps2K)		−**7.80**	3.59E-213	**2.11**	2.71E-65
**SPD_0327**	UDP-galactopuranose mutase (Cps2P)		−**8.45**	0	**2.03**	4.95E-61
**SPD_0328**	Glucose-1-phosphate thymidylyltransferase (Cps2L)		−**8.87**	0	**1.63**	3.58E-35
**SPD_0329**	Dtdp-4-dehydrorhamnose 3,5-epimerase (Cps2M)		−**9.34**	0	**1.68**	6.43E-39
**SPD_0330**	Dtdp-glucose 4,6-dehydratase (Cps2N)		−**8.54**	2.22E-303	**1.72**	1.71E-46
**SPD_0331**	NAD(P)-dependent oxidoreductase (Cps2O)		−**9.98**	0	1.37	3.72E-12
**SPD_0333**	Hypothetical protein		−**8.86**	1.24E-140	1.40	1.43E-07
Maltose, galactose, and host glycan sugar-specific utilization-related genes						
**SPD_289**	Bifunctional 2-keto-3-deoxy-6-phosphogluconate aldolase		−**8.42**	3.75E-98	**2.71**	6.91E-50
**SPD_290**	2-keto-3-deoxygluconate kinase		−**10.70**	1.40E-116	**2.57**	4.24E-57
**SPD_291**	Hypothetical protein		−**9.36**	3.49E-105	**2.98**	5.41E-53
**SPD_292**	Gluconate 5-dehydrogenase		−**8.30**	4.74E-60	**2.22**	2.80E-35
**SPD_293**	PTS *N*-acetylgalactosamine transporter subunit IIA		−**15.52**	2.62E-14	**1.91**	3.29E-10
**SPD_294**	Unsaturated chondroitin disaccharide hydrolase		−**28.12**	5.27E-53	**1.9**	4.04E-31
**SPD_295**	PTS *N*-acetylgalactosamine transporter subunit IIB		−**21.92**	1.31E-90	**2.37**	7.97E-48
**SPD_296**	PTS *N*-acetylgalactosamine transporter subunit IIC		−**27.79**	1.76E-87	**2.17**	1.21E-45
**SPD_297**	PTS *N*-acetylglucosamine transporter subunit IIBC		−**40.83**	1.75E-101	**2.09**	1.05E-51
**SPD_298**	Preprotein translocase subunit YajC		−**32.09**	2.67E-36	**1.99**	6.29E-22
**SPD_300**	Oligohyaluronate lyase		−**19.06**	7.34E-37	**1.93**	4.31E-22
**SPD_311**	Glucan 1,6-alpha-glucosidase		**6.53**	2.77E-80	−**1.91**	2.16E-43
**SPD_313**	S-ribosylhomocysteinase		**2.78**	3.43E-06	−**2.75**	5.94E-05
**SPD_1932**	Maltodextrin phosphorylase		**2.55**	0	−**6.38**	0
**SPD_1933**	4-alpha-glucanotransferase		**2.12**	0	−**6.78**	0
**SPD_1934**	Maltose/maltodextrin-binding protein		**2.82**	8.47E-215	−**2.52**	6.38E-94
**SPD_1935**	Sugar ABC transporter permease		**2.43**	8.81E-47	−**1.96**	8.43E-27
**SPD_1663**	Alpha,alpha-phosphotrehalase		**27.30**	9.72E-40	−**2.15**	1.69E-23
**SPD_1664**	PTS beta-glucoside transporter subunit EIIBCA		**8.25**	0	−**8.11**	4.12E-261
**SPD_RS4270**	Hypothetical protein		**2.32**	5.6E_03	−**2.12**	8.9E-03
**SPD_RS6165**	ABC transporter ATP-binding protein		−**2.43**	5.72E-89	**2.0**	6.87E-47
Miscellaneous genes	
**SPD_632**	Bifunctional phosphomethylpyrimidine kinase		−**2.99**	9.74E-90	**2.63**	5.55E-47
**SPD_890**	Hypothetical protein		−**2.41**	4.47E-208	**2.59**	1.79E-92
**SPD_1004**	NADP-dependent GAPDH		−**2.19**	3.46E-98	**1.9**	1.73E-50
**SPD_1166**	Hypothetical protein		−**2.17**	1.05E-129	**2.05**	7.02E-62
**SPD_1168**	Peptide ABC transporter permease		−**2.60**	2.27E-70	**1.90**	2.18E-38
Iron/ferrichrome transport genes
**SPD_1649**	Ferrichrome ABC transporter permease		**51.62**	1.24E-05	−**2.59**	2.32E-04
**SPD_1650**	Ferrichrome ABC transporter permease		**43.21**	5.69E-06	−**2.35**	9.74E-05
**SPD_1651**	Iron ABC transporter ATP-binding protein		**48.91**	2.35E-04	−**2.08**	2.58E-03
**SPD_1652**	Iron ABC transporter substrate-binding protein		**56.08**	1.01E-4	−**2.09**	2.067E-3

a Fold-change numbers in boldface indicate ≥1.5-fold or ≤−1.5-fold significant change. Numbers in regular font indicate ≤1.5-fold or ≥−1.5-fold insignificant change.

Further, 6 of 13 gene pairs encoding two-component systems showed differential regulation in individual or both D39ΔStkP and D39ΔPhpP mutants (Table S4A). For example, the transcript abundance of *rr08* (*spd_0081*/*saeR*) was upregulated only in D39ΔStkP (2.28-fold) but moderately in D39ΔPhpP (1.32-fold). Similarly, the expression of *rr10* (*vncS/R*, *spd_0525/spd_0524*) was downregulated (~3.0-fold) only in D39ΔPhpP but not in D39ΔStkP (~1.0 to 1.2-fold). The remaining four two-component systems (*rr03/liaSR-spd_0351/0352*, *rr13/blpHR*-*spd_0469/0468*, *rr05/ciaRH*-*spd_0702/0701*, and *rr12/comDE-spd_2064/2063*) were severely downregulated (2- to 31-fold) in both mutants (Table S4A).

**FIG 5:**
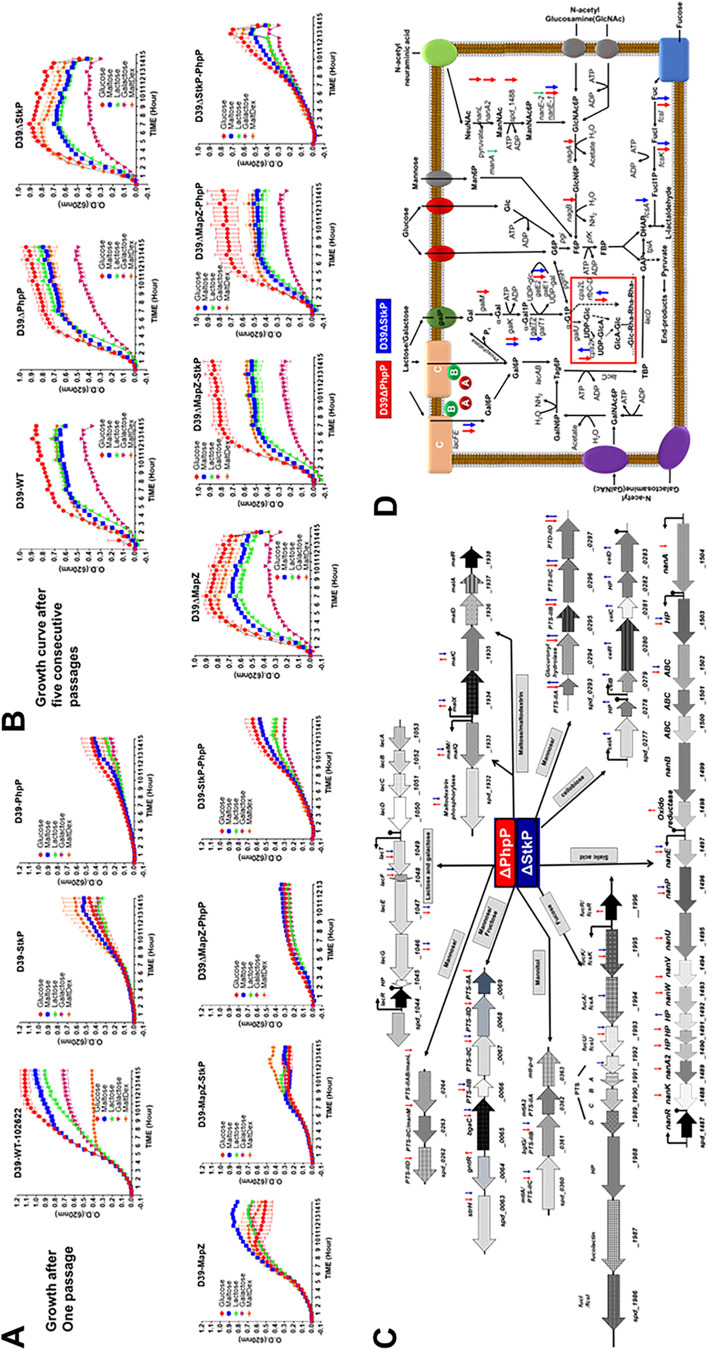
Growth curve profiles of S. pneumoniae D39-wild-type, the isogenic ΔPhpP and ΔStkP mutants, and corresponding wild-type *phpP/stkP*-complemented mutant strains. (A and B) Growth curves of pneumococcus strains were measured using chemically defined C+Y medium supplemented with different 1% (wt/vol) sugars as a sole carbon source (glucose, maltose, lactose, galactose, maltodextrin). Optical density (OD_620_) of growth at 37°C was measured every hour in microtiter plates for 15 h, as described in Materials and Methods. (A) Culture inoculum obtained from one passage, and (B) from 4 to 5 consecutive passages. Each data point represents an average reading obtained from three individually grown cultures measured in 3 wells using sterile 96-well U-bottom microtiter plates. Error bars represent mean ± SD. Growth curves in the presence of different sugars for each strain are color-coded as indicated in the inset of each panel. (C) PTS- and sugar transport-related genes found in specific operons regulated in the absence of PhpP (red) and StkP (blue). (D) Diagrammatic presentation of a pneumococcus cell showing sugar transporters as indicated in the cell membrane and cascade of metabolic pathways affected by the lack of PhpP (red) and StkP (blue). Inset with outlined square highlighting galactose metabolic pathways involved in polysaccharide capsule production. Up and down-facing red and blue arrows (C and D) represent up and downregulated genes. (see Table S1 to S4 for detail).

Concerning reciprocally regulated genes in these two mutants (Table 1), the differential regulation was essentially associated with the genes responsible for capsule production, carbohydrate metabolism, and iron/ferrichrome transport (Table 1). Particularly, while the transcript abundance of several genes of the capsule (*cps*) operon (*spd*_*0315* to *spd*_*0331*) was significantly increased in D39ΔStkP, the entire 17 *cps* genes displayed decreased transcript abundance by ~9 to18-fold (Table 1) in the D39ΔPhpP mutant. (see also Fig. 5D). Furthermore, the qRT-PCR analysis of capsule genes (*cps2A*, *cps2E*, and *cps2L*) also revealed decreased and increased mRNA transcript abundance in D39ΔPhpP and D39ΔStkP mutants, respectively, confirming that PhpP and StkP reciprocally regulate the transcription of capsule-related genes. Similarly, a matching transcript abundance profile for an additional eight genes by qRT-PCR validated RNA-seq analysis in general (Table 2).

**TABLE 2:** qRT-PCR-based confirmation of the RNA-Seq-based transcriptome analysis of the D39ΔPhpP and D39ΔStkP mutants^*a*^

SPD_#ORF	Gene symbol/ function	D39ΔPhpP vs D39-WT			D39ΔStkP vs D39-WT		
		Normalized linear ratio	Normalized fold change	RNA-Seq fold change	Normalized linear ratio	Normalized fold change	RNA-Seq fold change
1543	*phpP*	3.6 × 10^−6^ ±1.97 × 10^−6^	−**370,370**	−**449.4**	2.23 ± 0.089	**2.23**	−1.23
1542	*stkP*	1.01 ± 0.07	1.2	−1.8	4.44e-5 ± 2.5e-5	−**22,522**	−**2,809.18**
63	*strH*	0.14 ± 0.0025	**−7.47**	**−21.47**	0.041 ± 0.008	−**24.4**	−**2.00**
301	*regR*	0.20 ± 0.007	**−5.0**	**−3.5**	1.15 ± 0.06	1.15	−1.05
315	*cps2A*	0.11 ± 0.004	**−9.1**	**−14.73**	7.81 ± 0.29	**7.81**	**1.5**
319	*cps2E*	0.09 ± 0.0006	**−10.9**	**−15.13**	8.21 ± 0.75	**8.21**	**1.56**
328	*cps2L*	0.37 ± 0.01	**−2.8**	**−8.87**	12.17 ± 0.98	**12.17**	**1.63**
1046	*lacG2*	0.10 ± 0.001	**−10**	**−4.69**	0.04 ± 0.001	−**23.8**	−**3.43**
1504	*nanA*	0.11 ± 0.001	**−8.77**	**−4.12**	0.054 ± 0.002	−**18.5**	−1.09
1633	*galT2*	0.66 ± 0.02	**−1.51**	**−1.8**	0.042 ± 0.002	−**23.0**	−**2.6**
1797	*ccpA*	1.39 ± 0.062	1.39	−1.27	1.08 ± 0.07	1.1	−1.46

a Numbers in boldface indicate a significant change. Numbers in regular font indicate insignificant change. #ORF-Open reading frame number.

### PhpP/StkP positively influences pneumococcal carbohydrate metabolism and contributes to metabolic fitness.

Pneumococci use glucose as the primary carbon source for growth and energy. In addition, S. aureus Stk1-mediated phosphorylation of CcpA modulates CcpA-regulated carbohydrate metabolism (67), a master regulator of carbohydrate metabolism (68). Since the pneumococcus CcpA is essential to ensure optimal metabolic fitness in S. pneumoniae (53), we hypothesized that the mutant strains lacking StkP or PhpP would display altered growth patterns when grown in different sugars.

The reduced growth of D39ΔPhpP mutant in the presence of nonglucose sugars in the initial passage indicated that the uncontrolled StkP-mediated phosphorylation in the absence of PhpP might adversely affect the bacterial ability to grow in the presence of nonglucose sugar (Fig. 5A). Noticeably and as reported previously for nonencapsulated pneumococcal strain grown in the glucose-containing medium (69), different pneumococcal mutants, including double mutants lacking MapZ-StkP, MapZ-PhpP, and StkP-PhpP showed improved growth after 4 to 5 sequential passages (Fig. 5B). However, the growth pattern of D39ΔStkP-ΔPhpP in the presence of glucose and different nonglucose sugars did not improve significantly (Fig. 5A and B). Transcriptome analysis of D39ΔStkP and D39ΔPhpP mutants for “carbohydrate metabolism and transport” genes revealed downregulation of most genes belonging to the lactose, galactose, fructose, cellobiose, maltodextrin, mannose, mannitol, and fucose metabolism and phosphotransferase system (PTS) (Fig. 5C, Table S4B), supporting the observed growth retardation of the mutants in nonglucose sugars. Noticeably, the expression of the gene encoding CcpA (catabolite control protein), the master regulator of sugar metabolism (53, 68), and its coregulator, HPr, and HPr kinase/phosphorylase did not show any significant changes in D39ΔPhpP and D39ΔStkP mutants (*ccpA*/*spd_1797*, −1.25_ΔPhpP_/-1.46_ΔStkP_-fold; *ptsH/hpr*/*spd_1040*, −1.13_ΔPhpP_/−1.67_ΔStkP_-fold; and *hprK/P/spd_1244*, −1.17_ΔPhpP_/1.04_ΔStkP_-fold). Based on these results showing modulated functional expression of several genes, including those encoding the proteins involved in the carbohydrate metabolism of D39ΔStkP and D39ΔPhpP mutant strains, we hypothesized that posttranslationally modified CcpA could be responsible for the observed changes.

### StkP and PhpP reversibly phosphorylate CcpA.

**FIG 6:**
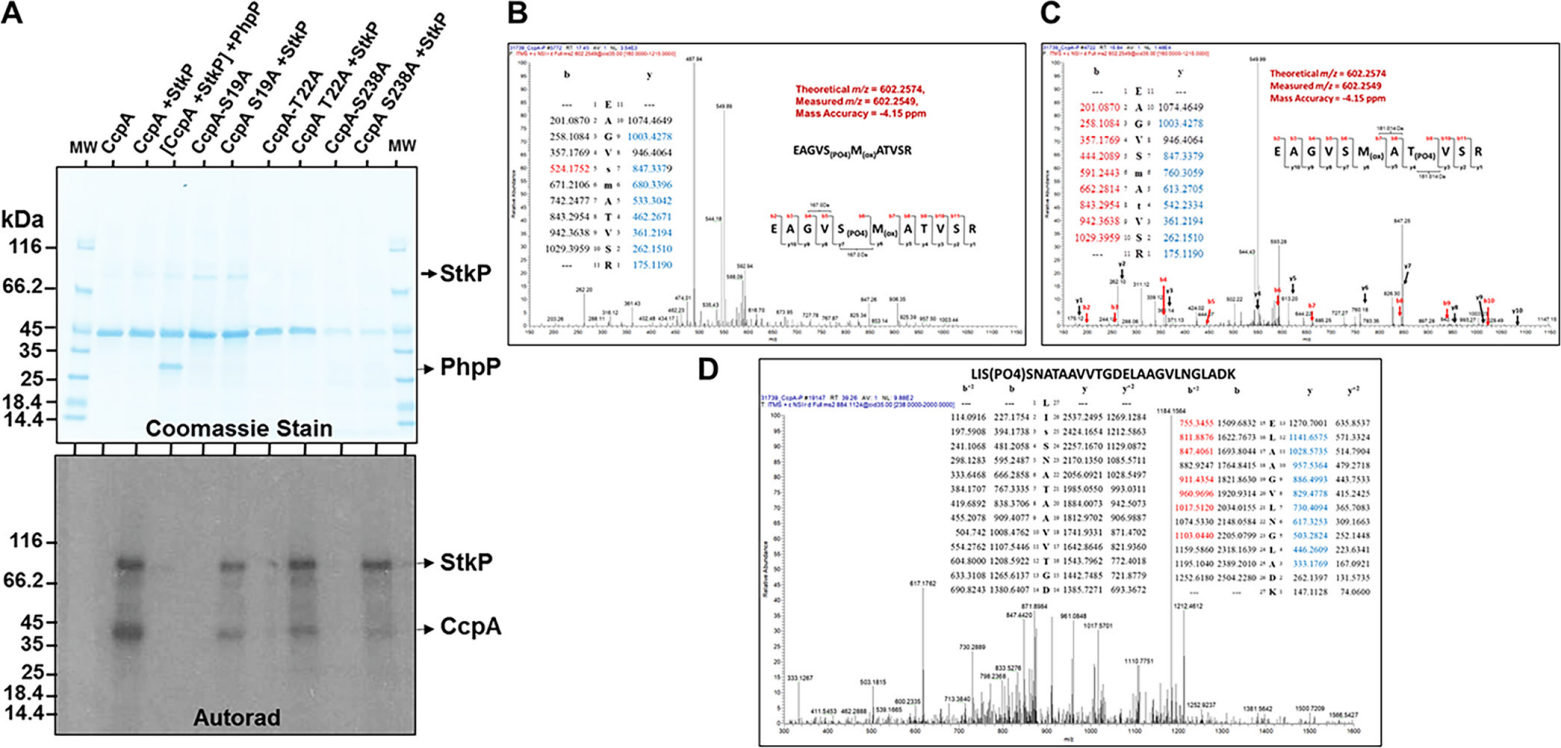
Reversible *in vitro* phosphorylation of CcpA by StkP and PhpP. (A) *In vitro* phosphorylation of CcpA and its mutated variant derivatives CcpAS19A, CcpAT22A, and CcpAS238A in the presence of StkP. The dephosphorylation of CcpA-P^32^ was carried out in the presence of PhpP. As indicated and visualized by autoradiography, reversible phosphorylation was carried out in the presence or absence of γ^32^P-ATP and StkP/PhpP. The upper and lower panels are the same gel visualized with Coomassie stain followed by autoradiography. (B, C, and D). Mass-spectrometric analysis of *in vitro and in vivo* StkkP-phosphorylated CcpA. SDS-PAGE-resolved *in vitro* and *in vivo* phosphorylated CcpA protein bands were excised and trypsinized. The resulting tryptic peptides were subjected to LC-MS/MS analysis as described in the Materials and Methods section. LC-MS/MS profiles of the two 11-mer peptides ([B] EAGVS^19^MATVSR, and [C] EAGVSMAT^22^VSR). Identified phosphosites, S19 and T22, within these peptides *in vitro* and *in vivo* phosphorylated CcpA is shown in the respective insets. (D) S238 residue in a 27-mer peptide (LIS^238^SNATAAVVTGDELAAGVLNGLADK) was identified as a minor phosphosite, primarily *in vitro* phosphorylated CcpA.

The above results and published reports showing the possible direct and indirect role of CcpA in influencing the expression of pneumococcal capsule genes (51, 53, 70), prompted us to examine whether StkP/PhpP-mediated reversible phosphorylation exerts its effects via posttranslational modification of CcpA. In the radioactivity-based *in vitro* phosphorylation assays, StkP and PhpP reversibly (phosphorylation and dephosphorylation, respectively) phosphorylated recombinant CcpA (Fig. 6A). Additionally, to determine the *in vivo* phosphorylation of CcpA by StkP, the kinase domain of StkP (StkkP) and His-tagged CcpA were expressed in the pCDFDuet vector and D39ΔCcpA::*his-ccpA* complemented strain. The *in vivo* phosphorylated purified, and SDS-PAGE resolved CcpA protein bands were subjected to mass-spectrometry analysis and compared with nonphosphorylated and *in vitro* StkP-phosphorylated (using cold ATP) CcpA. The comparative analysis of *in vitro* (using cold – ATP) and *in vivo* phosphorylated CcpA proteins revealed Ser19 (*In vivo and in vitro*), Thr 22 (*in vitro* and *in vivo*), and S238 (*in vitro*, with low probability) as three major phosphosites in phosphorylated CcpA (Fig. 6B). Subsequently, three variant forms of the recombinant CcpA protein, CcpAS19A, CcpAT22A, and CcpAS238A, were subjected to *in vitro* phosphorylation by StkP. Each of these variant proteins showed substantially decreased phosphorylation (Fig. 6A), indicating that all three residues contribute to the StkP-mediated CcpA phosphorylation.

### StkP-phosphorylated CcpA does not efficiently bind to the *cps2A* promoter.

**FIG 7:**
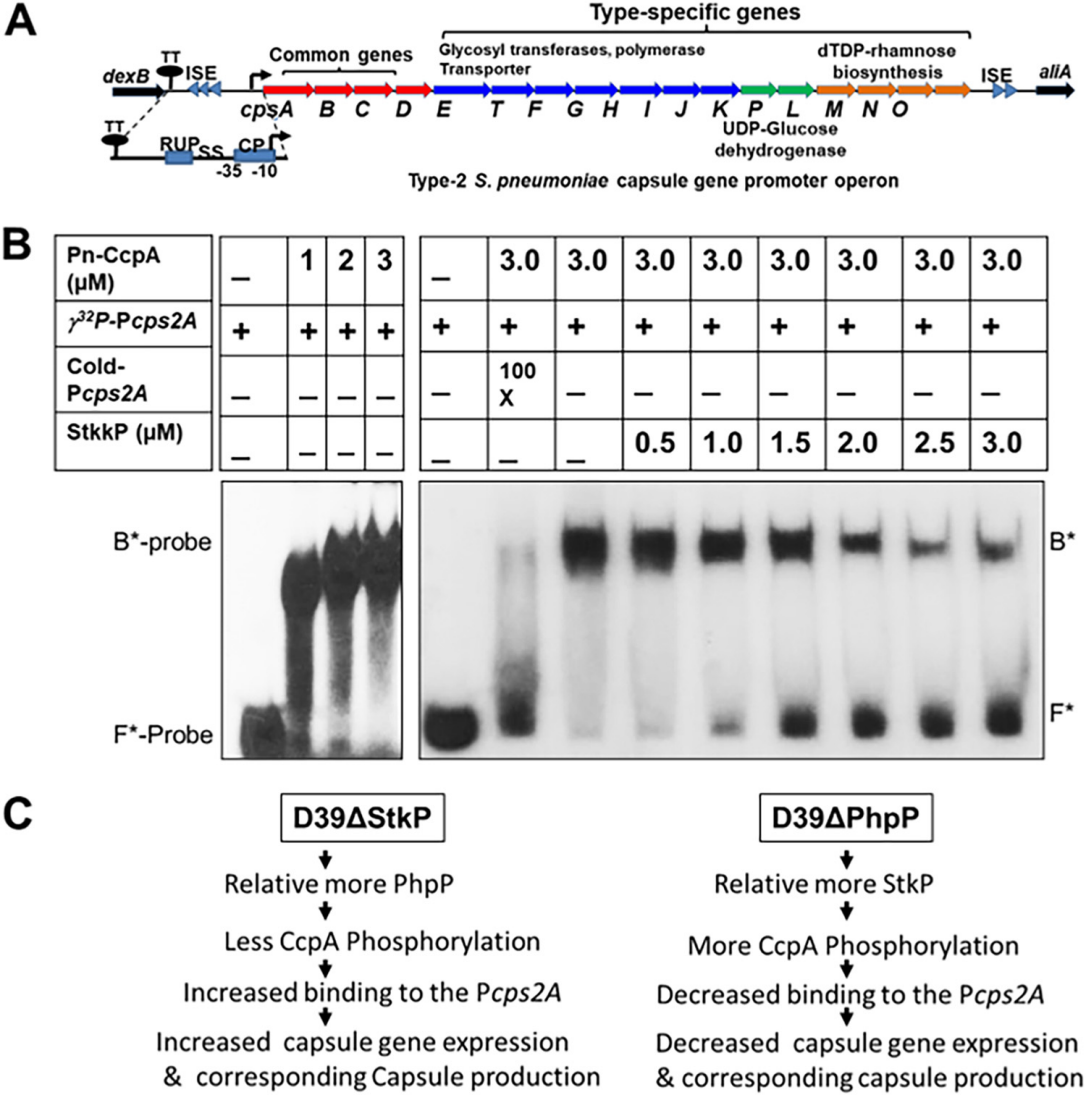
The impact on the binding of *in vitro* StkP-phosphorylated and nonphosphorylated CcpA to the *cps2A* promoter. (A) The diagram shows that the cps2 operon contains 17 genes and an upstream promoter region located between *dexB* and *aliA* genes. The functional nature of promoter region encompasses transcription terminator (TT), insertional element (ISE), repeat region RUP, spacing sequence (SS), and core promoter (CP). (B) Electrophoretic mobility shift assay (EMSA) for StkP-phosphorylated and nonphosphorylated CcpA was conducted using *cps2A* as the binding probe. Left panel, EMSA was performed using a ^32^P-labeled fragment (–250/+35) with an increasing (1 to 3 μM) amount of CcpA. Right panel, EMSA was performed using a ^32^P-labeled probe, the promoter probe. A sequence of the 5′ upstream (–250/+35) region of *cps2A* was used with a constant amount of CcpA and increasing amounts of StkP (0.5 to 3 μM, lanes 4 to 9). Lanes 1 and 2, EMSA was performed using a ^32^P-labeled probe (10,000 dpm) in the absence (lane 1) or presence (lane 2) of 100× excess amount of cold probe as a competitor to confirm the specific DNA/protein complex. Electrophoresis of the ^32^P-labeled probe alone is shown in the first lane of each panel. Results represent three similarly conducted independent experiments. B* represents bound form with retarded migration of the labeled probe. F* represents unbound, fast-migrated, free probe. (C) Impact of the repressor activity of StkP-mediated phosphorylation on the transcription of *cps2* genes and the observed regulation of the polysaccharide capsule formation.

The reciprocal expression levels of the polysaccharide capsule, along with the corresponding transcript abundance of *cps2* genes in D39ΔPhpP and D39ΔStkP mutants and the ability of CcpA to bind to the *cre* locus within the *cps2A* promoter (51, 70, 71) suggested that the StkP-mediated phosphorylation of CcpA might adversely affect this binding. Upon examining the CcpA binding to the ^32^P radiolabeled *cps2A* promoter by EMSA, we observed that CcpA interacted with the *cps2A* promoter and shifted its migration dose-dependently (1 to 3 μM), as reported previously (70). However, the *in vitro* phosphorylated CcpA in the presence of increasing amounts of StkkP (0.5 to 3.0 μM) displayed a dose-dependent reduced binding to the P*cps2A* probe, and proportionately increased amounts of the nonmigrated labeled promoter probe (Fig. 7). These results thus indicated that the binding of CcpA to the P*cps2A* promoter and the observed differential transcript abundance of capsule operon genes and corresponding capsule production in D39ΔStkP and D39ΔPhpP mutants were inversely proportional to the StkP-mediated CcpA phosphorylation.

### PhpP and StkP play an important role in the modulation of S. pneumoniae virulence.

While the pneumococcus capsule is known to be the major virulence factor, several other virulence factors also play a crucial role in pneumococcal adherence to and invasion of nasopharyngeal cells and hence in the virulence (12, 14, 72). Along with differential transcript abundance of capsule biosynthesis-related genes, seven genes (*blpU*, *strH*, *pspA*, *endoD*, *nanA2*, *nanA*, and *spd0335*) of the 16 differentially regulated potential virulence genes in the D39ΔStkP and D39ΔPhpP and virulence-regulating two-component systems were downregulated (Table 1, Table S4A). These results prompted us to investigate whether differentially regulated virulence genes participated in pneumococcal virulence.

**FIG 8:**
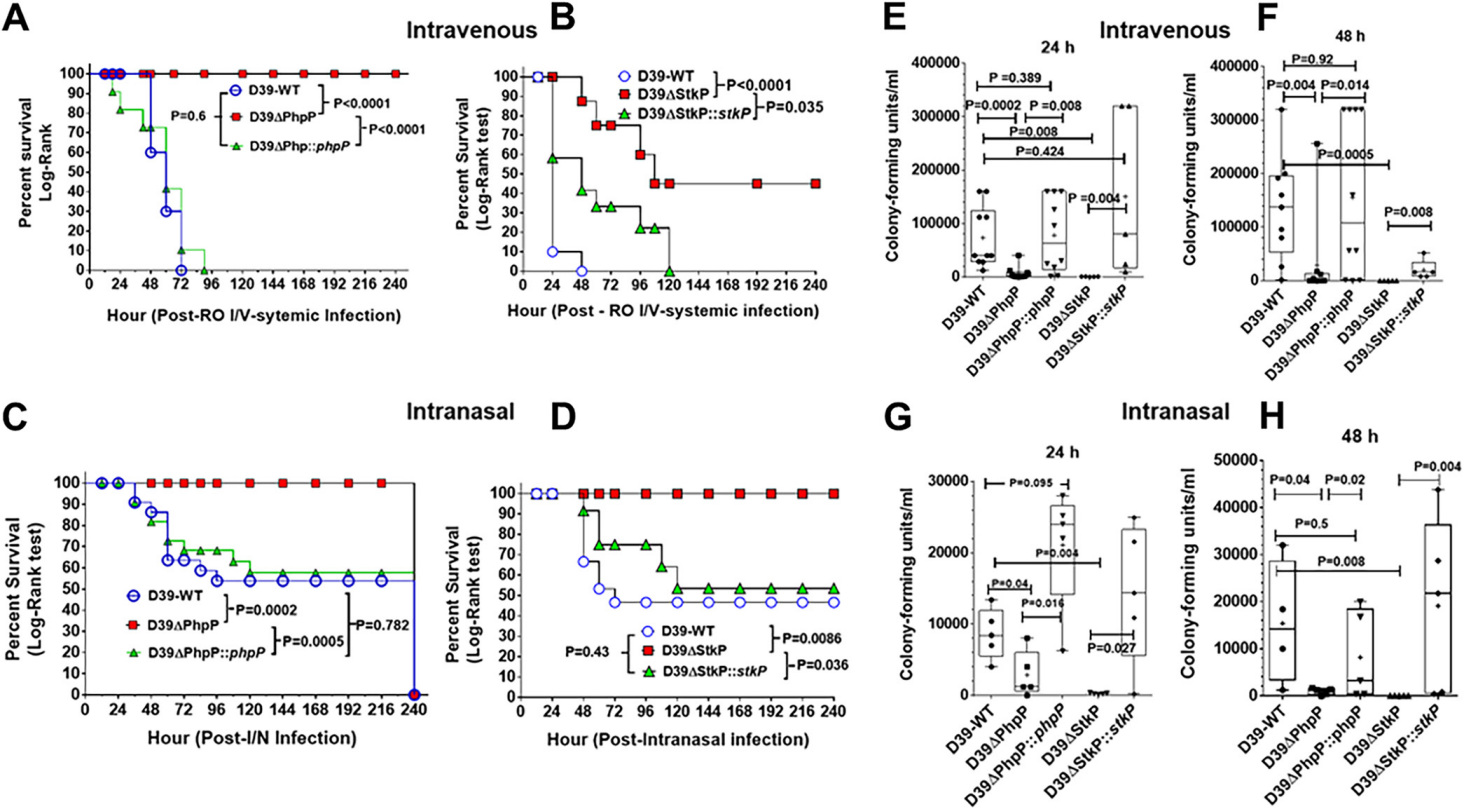
Role of PhpP and StkP in the modulation of pneumococcal virulence. Effects of the deletion of the *phpP* and *stkP* genes on pneumococcal virulence as measured by challenging mice retroorbitally (A and B) (5 × 10^3^ CFU/100 μL/mouse) and intranasally (C and D) (5 × 10^7^ CFU/20 μL/mouse) with pneumococcus strains (D39-Wt, isogenic ΔPhpP/ΔStkP mutants, and corresponding *phpP*/*stkP*-complemented strains) in mouse septicemia and colonization infection models. Panels A to D show combined results (a group of 10 and 7 mice) on survival/mortality of mice injected retroorbitally/intranasally with pneumococcal strains and observed for 10 days. Individual survival/mortality curves are color-coded as indicated in the panel inset. Percent survival/mortality patterns observed throughout 10 days were plotted by the Log-Rank test using GraphPad Prism 6 software. (E–H) CFU of the wild-type, mutant, and complemented strains in blood were determined after 24 h and 48 h of systemic (E and F) and intranasal (G and H) infection. The horizontal line within the bars indicates the median CFU from 4 to 10 mice (systemic disease) or five mice (intranasal infection). *P* values of significance shown in panels A to H were calculated using GraphPad Prism 6. *P* < 0.05 was treated as a significant difference.

To that end, we examined the virulence potential of D39ΔPhpP and D39ΔStkP mutants compared to their parental wild-type and the complemented strains employing mouse infection models for septicemia and nasopharyngeal colonization/lung infection. The results of the septicemia model revealed the complete attenuation of virulence in the D39ΔPhpP mutant strain as all mice survived for 10 observation days (Fig. 8A) and even when observed after 3 weeks postinfection (data not shown). In contrast, mice infected similarly with D39ΔStkP displayed attenuation with only 50% mortality, possibly due to the increased production of the capsule (Fig. 7B). Mice infected similarly with the wild-type and the *phpP*- or *stkP*-complemented strains died within 2 to 5 days postinfection (Fig. 8A and B). A similar pattern of virulence attenuation was also observed when the mice were infected by the intranasal route using 2.82 log_10_ higher inoculum (5 × 10^7^ I/N versus 7.5 × 10^4^ I/V CFU/animal) of D39ΔPhpP (Fig. 8C) and D39ΔStkP mutants (Fig. 8D). However, ~47% of infected mice (7 [D39ΔStkP] and 8 [D39ΔPhpP] out of 17 mice) belonging to the wild-type and complemented groups still survived. Irrespective of the route of infection, the inclusion of two different infection models consistently demonstrated that the D39ΔPhpP mutant, which showed significant decreases in the capsule formation without displaying significant growth defects in the enriched media, was attenuated for virulence. The D39ΔStkP mutant, on the other hand, despite its growth defect, showed relatively more virulence during systemic infection.

Consistent with the virulence pattern described above, the median pneumococcal CFU counts measured at 24 h, and 48 h in the blood of the mice retroorbitally infected with the D39ΔStkP/PhpP mutant strains were found to be significantly low in comparison to those in the wild-type and corresponding complemented strain-infected mice (Fig. 8E and F). These results indicated that the mutant strains could not survive in the blood and were phagocytosed and killed readily by innate host immune responses. In contrast, the wild-type and the complemented strains survived and multiplied (Fig. 8E and F). We also observed a similar trend at 24 h and 48 h when we counted CFU in the blood of mice infected intranasally with these strains. However, the total number of CFU was relatively low in the mutant-infected mice, and 5 of 17 survived at the end of the observation period (Fig. 8G and H).

Along with these findings, the Gram-staining of the tissue sections obtained from the lungs of the mice infected with the D39ΔPhpP mutant strain revealed the absence of any discernible bacterial colonization of the lung tissues (Fig. S3). In contrast, the lumen and the interstitial spaces of the lung alveoli of the wild-type- and the *phpP*-complemented strain-infected mice showed a profuse number of bacteria in the lumen (Fig. S3). Furthermore, the histopathology (hematoxylin and eosin/H and E staining) of the sections of lung tissues obtained from the wild-type and complemented strain-infected mice also revealed thick inflamed alveolar walls compared to the lungs of D39ΔPhpP mutant-infected mice exhibiting thin-walled alveoli with much larger lumen space (Fig. S3). The histopathological pattern of D39ΔStkP and corresponding complemented strains mimicked similarly (data not shown).

### D39ΔPhpP mutant evokes distinct protective inflammatory responses in the lung epithelial cell culture.

**FIG 9:**
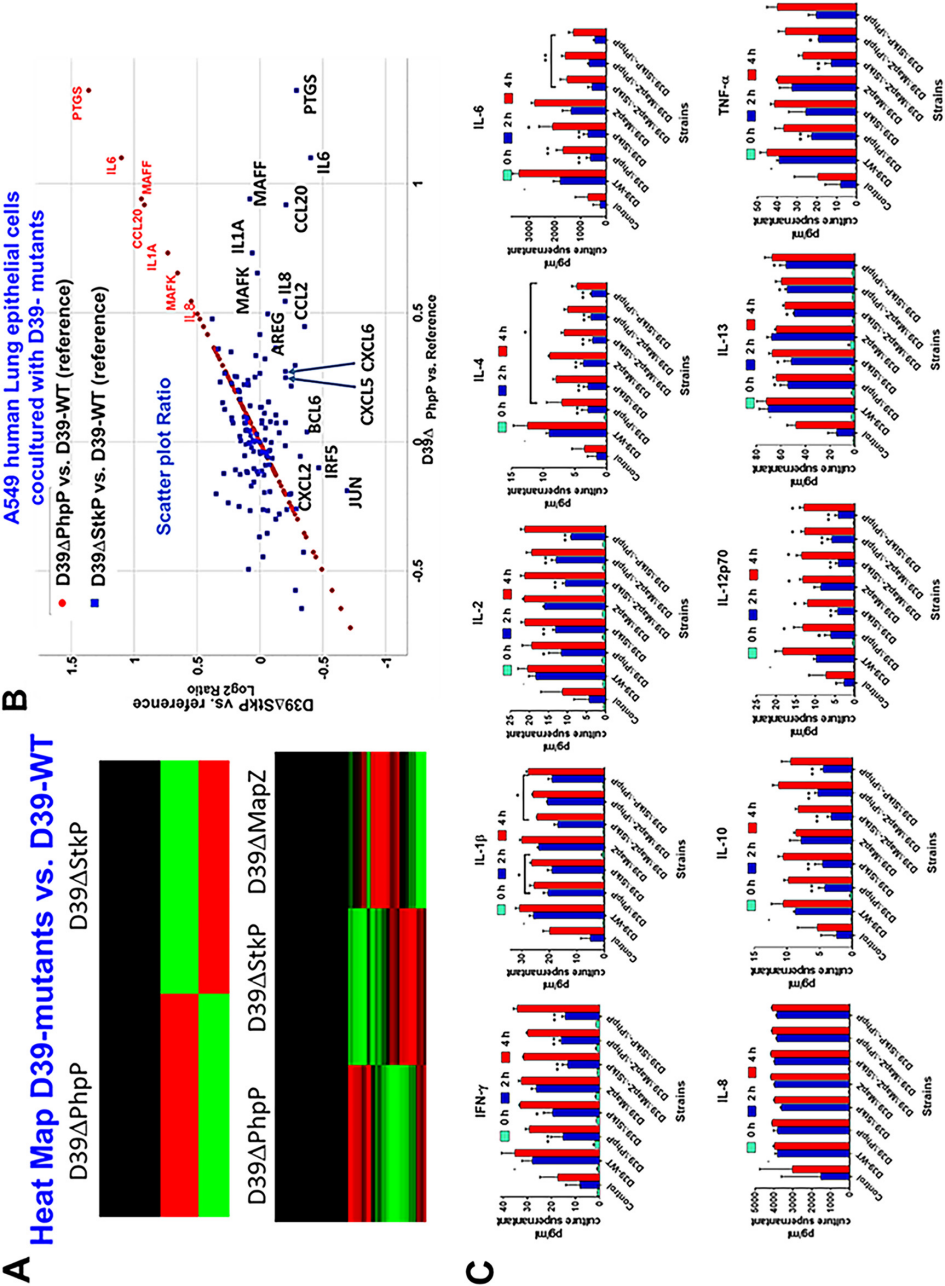
Evaluation of proinflammatory gene expression and quantitative chemokine profiling of A549 human lung tissue culture cells cocultured with D39-WT and isogenic mutants. High-quality total RNA was extracted from A5459 human lung epithelial cells after coculturing for 4 h with D39-WT, D39ΔStkP, and D39ΔPhpP and D39ΔMapZ mutants (MOI 50:1) in six-well plates. The extracted RNA samples from the A549 cells were then subjected to mRNA expression of 255 established inflammatory genes as described and recommended by NanoString Technologies. The data were analyzed by nCounter version 3.0 software. *P* < 0.05 was treated as a significant difference.(A) The heatmap shows the relative expression of inflammatory genes in A549 cells cocultured with D39-derived mutants versus D39-WT. (B) The scattered plot ratio diagram demonstrates differentially expressed inflammatory genes in A549 cells cocultured with D39ΔPhpP (brown circle) and D39ΔStkP (blue square) compared with D39-WT. Seven inflammatory genes shown in red fonts are relatively highly expressed (1.5 to 3-fold) in the presence of D39ΔPhpP compared to D39ΔStkP. (C) Meso Scale Discovery (MSD)-based quantitative determination of chemokines secreted in the supernatant of A549 cells cocultured with the D39-WT, D39ΔPhpP, D39ΔStkP, and D39ΔMapZ single mutant strains, and the D39ΔMapZ-ΔStkP, D39ΔMapZ-ΔPhpP, and D39ΔStkP-ΔPhpP double mutant strains. Coculture supernatants were collected at 0 h, 2 h, and 4 h and subjected to MSD chemokine analysis as described in the Materials and Methods. As listed, 10 chemokines were tested against three individual cultures for each mutant. Each error bar denotes an average reading ± SD. Results were statistically analyzed by Student's *t* test using GraphPad Prism 6. All comparisons within individual chemokines at different hours are with values obtained with D39-WT at a specific time interval. *, *P* < 0.05; **, *P* < 0.01. Error bars without asterisk marks indicate no significant difference compared to D39-WT at specified time intervals.

To further dissect whether in addition to the pneumococcal division process, its growth defect, and the presence or absence of the polysaccharide capsule *per se*, other host factors are responsible for pneumococcus virulence, we cocultured D39-WT and isogenic mutant strains D39ΔStkP, D39ΔPhpP, and D39ΔMapZ with A549 human lung cells for 4 h and determined the impact of bacterial-cell interactions on the expression of 255 inflammation-related genes employing NanoString technology. We examined unamplified mRNA copy numbers of 255 inflammatory genes in one sample to obtain multigene differential expression profiling. Out of 255 genes, only 48 genes showed significant difference (*P* < 0.05) in A549 cells while in contact with these mutants (19 with D39ΔStkP, 32 with D39ΔPhpP, and 11 with D39ΔMapZ) (Table S5A and B) compared with the wild-type D39-Wt strain (Fig. 9A). Upon further analysis of the results of these mutants’ interaction with host cells, the heat map of these differentiated genes in A549 cells showed distinct virulence phenotypes. (Fig. 9B, Table S5B). Particularly, A549 cells cocultured with D39ΔPhpP in comparison to D39ΔStkP showed upregulated mRNA expression (1.5- to 3-fold) of 9 inflammatory genes (FOS-1.5, MAFK-1.55, IL1A-1.59, IL-8-1.67, CCL2-1.74, MAFF-1.55, CCL20-2.18, IL-6-2.8, and PTGS2-3.1) (Fig. 9B, Table S5B sheet 1).

In a separate similar experiment, tissue culture supernants from cocultured A549 cells with different D39-derived mutants were collected at 0, 2, and 4 h and examined quantitatively for the secretion of IFN-γ, TNF-α, and 8 interleukins by multiplex electrochemiluminescence (ECL)-based meso scale discovery (MSD) cyotkine/chemokine analysis (Fig. 9C). These results revealed significantly less amounts (*P* < 0.05, *P* < 0.01) of most chemokines in the supernatants of A549 cells cocultutred with D39ΔPhpP and D39ΔStkP versus D39-WT and D39ΔMapZ collected after 2 h of incubation. The double mutants lacking MapZ and StkP or PhpP, and lacking StkP and PhpP showed similar low expression levels at 2 h. The significantly dampened critical inflammatory chemokine responses against D39ΔPhpP and D39ΔStkP in comparison to D39ΔMapZ and D39-WT supports the observed virulence attenuation of D39ΔPhpP mutant and reduced virulence of D39ΔStkP in comparison to D39-WT (Fig. 8).

PhpP and StkP together, thus, served as a critical cognate enzyme pair to control pneumococcal cell division, virulence, and metabolic fitness by direct or indirect modifications of transcription factors such as CcpA and subsequently transcription of genes required for polysaccharide capsule formation and other metabolic/transport genes. In addition, the mutants' differential cell surface structures and ability to grow in the glucose or nonglucose sugar-enriched host environment also modulate the host- inflammatory genes during infection. Thus, StkP- and PhpP-mediated posttranslational modifications play a crucial role in the modulation of pneumococcal virulence and disease pathogenesis. Given this multimodal functionality, both StkP and PhpP can serve as promising therapeutic targets to counteract encapsulated and nonencapsulated pneumococcal diseases.

## DISCUSSION

Pneumococcal colonization of the nasopharynx is a prerequisite for the subsequent steps involving bacterial adherence and persistence for the disease commencement. Although the polysaccharide capsule is the major virulence factor, other noncapsule factors, including several surface proteins, also have been incriminated in pneumococcal adherence, invasion, and dissemination (6). Pathogenic mechanisms underlying pneumococcal diseases, like many other Gram-positive pathogens, are thus multifactorial (6, 73, 74). Among many virulence regulators, the role of Ser/Thr kinase (StkP) also has been incriminated in pneumococcal virulence primarily as a regulator of the fundamental growth process, i.e., cytokinesis (28, 39). The PhpP serves as a counteracting or balancing factor in the homeostasis of cytokinesis. However, the role of StkP and PhpP, unlike similar homolog enzymes of other Gram-positive pathogens (36, 43, 75, 76) in other cellular activities, has not been well recognized. Published microarray analyses of noncapsulated pneumococcus-derived mutant lacking StkP has revealed differential expression of genes that encode proteins involved in cell wall metabolism, pyrimidine biosynthesis, DNA repair, iron uptake, and oxidative stress (77). In the present study, we provide an essential and first insight into the global regulatory role of PhpP and StkP in the encapsulated pneumococcus pathophysiology. Considering several advantages of RNA-seq-based transcriptome analysis (78) over the limitations of microarray analysis (79), we opted to carry out RNA-seq-based analysis of the ΔPhpP and ΔStkP mutants derived from the encapsulated pneumococcus strain D39. The major highlights of the present investigation are the revelation of the mechanism underlying the modulation of the polysaccharide capsule formation and the metabolism genes involved in the building blocks of pneumococcal capsules. (Table 1, Table S2). The additional highlights of the comparative global transcriptome analysis of D39ΔStkP and D39ΔPhpP mutants included 45 to 50% of the DEGs unique to be regulated by either StkP or PhpP, and 29% DEGs were are under the control by both enzymes. The observed direct and indirect regulations indicate that although StkP and PhpP have reciprocal biochemical functions, their physiological existence seems to be mutually exclusive as a monocomponent regulatory system and may interact with other corresponding cross-reacting counteracting kinase or phosphatase enzymes. Results obtained from the present transcriptomic study showing modulated expression of many genes could also be the direct and indirect of the reversible phosphorylation of the gene products as described recently in two phosphoproteomic analyses of ΔPhpP and ΔStkP mutants derived from the nonencapsulated S. pneumoniae *strains* (26, 27). Although these analyses did not show the implications of the observed *in vivo* phosphorylation in a mechanistic form, our study complements the published phosphoproteomic analyses to quite an extent.

In the present study, transcriptome analysis of the D39ΔPhpP mutant versus D39-WT revealed the precisely targeted functional category of genes responsible for metabolic fitness and virulence. Pneumococcus lacks the respiratory chain or tricarboxylic acid cycle for energy production, leaving glucose as the primary source of energy (65). The ability to survive and thrive in various host niches endowed with physiologically diverse microenvironments and typically deficient in glucose and other essential cation contents reflects that the pneumococcus possesses a regulatory mechanism to utilize other sugars as an alternative carbon source (80). In the upper and lower respiratory tracts, mucins are major components of the mucus that cover the epithelial surfaces (81). Mucins are heavily O-glycosylated glycoproteins and composed of *N*-acetylglucosamine (GlcNAc), *N*-acetylgalactosamine (GalNAc), *N*-acetylneuraminic acid/sialic acid (neuNac), galactose, fucose, and sulfated-sugars linked to the core protein (82–84). Sialic acid also plays a dynamic role in pneumococcal adhesion and invasion and as a molecular signal for forming biofilms, nasopharyngeal colonization, and invasion (85, 86). The major content of human saliva is also sialic acid (84). Galactose is a critical sugar in pneumococcal colonization and infection (56). Pneumococcus relies and thrives on these glycan-derived sugars. Pneumococcus is endowed with more than 20 phosphotransferase systems (PTS) to properly transport various complex sugars (80). Besides some sugar-specific PTS, many PTS are responsible for transporting more than one sugar, and some sugars, such as galactose, may use more than one PTS (80). Given such complexity, the ability of PhpP and StkP to influence the expression of several PTSs (Table S4B) in a similar manner indicates that StkP-PhpP, individually and as a pair, play a decisive role in this complex metabolic regulation, and ultimately in pneumococcal colonization (56). Since the key genes involved in galactose metabolism (*spd_1634/galK*, *spd_1633/galT*, and *spd_1635/galR*, and *spd_1648/galE*) were downregulated (2 to 4-fold) (Table S1, Fig. 5A to D) in both D39ΔStkP and D39ΔPhpP mutants, the growth of these mutants in the presence of galactose could not be sustained beyond an OD of 0.2 to 0.4. The recent proteomic studies of nonencapsulated D39 strain-derived PhpP and StkP mutants also have observed differential expression and increased phosphorylation of PTS proteins, especially in galactose operon (26, 27). The mechanism underlying protein phosphorylation affecting PTS gene expression is presently unknown. These results, however, support our observation that none of the mutants lacking StkP, PhpP, and/or MapZ could grow efficiently in the culture media containing nonglucose sugars, especially in the presence of galactose. Although presently unknown, the mechanism of this downregulation is an important field of future research as galactose metabolism is physiologically linked to polysaccharide capsule production (Fig. 5D).

Catabolite control protein (CcpA) enables pneumococcus (53) and many other Gram-positive pathogens (67, 68, 87–90) to utilize nonglucose carbohydrate sources and survive in the glucose-depleted host microenvironment. CcpA thus plays an essential role in maintaining pneumococcal metabolic fitness and virulence. It also plays an indirect role in polysaccharide capsule production in certain Gram-positive bacteria, including pneumococci (53, 55, 91), and partially regulates galactose metabolism (53, 91). In the present study, however, we observed the downregulation of genes controlling several nonglucose sugar metabolism and transport but not that encoding CcpA. These results indicated that the functionality of CcpA, i.e., its binding ability to the “*cre*”-region containing promoters, might have been altered, resulting in compromised metabolic fitness in the absence of PhpP and StkP. (Fig. 7). Based on the differential expression of proteins involved in sugar metabolism, a recent proteomic analysis study of pneumococcus mutants lacking StkP and PhpP made a similar prediction (27). However, StkP in this study did not seem to target CcpA. A report showing the abrogation of the binding ability of the S. aureus Stk1-phosphorylated CcpA to the “*cre*” promoters, such as *ccpA*, *citZ*, *tst*, *pckA*, *ald*, and *malR* indicated that the Stk1-mediated phosphorylation in S. aureus potentially modulates the CcpA repressor activity toward the target genes (67). To understand how capsule assembly is coordinated with cell wall biosynthesis in S. aureus, a recent report has shown that S. aureus Ser/Thr kinase, PknB senses cellular lipid level II levels and negatively controls capsule synthesis based on proteomic analysis and showed increased capsule production in the absence of PknB (59). The study on LytR-CpsA-Psr (LCP) glycopolymer transferases, especially CpsA, has shown that they play an essential role in the attachment of capsule assembly to the cell wall (92). While the study on the S. aureus capsule has focused on the capsule biosynthesis purely on proteomic and biochemical levels (59), our study has highlighted that StkP and PhpP modulate pneumococcal capsule biosynthesis by reciprocal expression levels of genes responsible for the production of building blocks of the polysaccharide capsule. At present, it is not known whether CpsA or other LCP motif-containing enzymes serve as the substrate for StkP in pneumococcus or if there is cross phosphorylation/dephosphorylation between StkP/PhpP and Cps2D (BY-tyrosine kinase)/CpsB (Tyrosine phosphatase) or non-BY (bacterial Tyrosine) kinase (Ubk/ubiquitous bacterial kinase) (93) or PTPs (protein tyrosine phosphatases). This knowledge gap can be attributed to the fact that previous studies on StkP and PhpP/UbK are carried out using nonencapsulated pneumococcus strains to focus primarily on their cell division, formation of the ovo-coccoid morphology and mechanisms of cytokinesis. Such cross-phosphorylation phenomena are reported in Gram-positive bacteria such as Bacillus subtilis (94–96) and S. pyogenes (64, 97). Recently, pneumococcal VncR has been reported to regulate capsule polysaccharide synthesis, although in a strain-specific manner (52). VncR is the response regulator of the vancomycin resistance locus (*vncRS* operon or *rr10/spd_0524/0525*). In the absence of PhpP, but not in the absence of StkP, both *vncRS* genes were 3-fold downregulated, supporting the downregulated capsule formation in D39ΔPhpP. Whether the regulation of polysaccharide capsule formation by CcpA through reversible phosphorylation StkP/PhpP also occurs through VncRS/RR10 TCS is presently unknown. ComE, an essential response regulator negatively regulates the expression of capsular polysaccharide locus (98). Both *comED*/*rr12*/*spd2063-2064* expression is severely downregulated in D39ΔStKP and D39ΔPhpP mutants (20 to 30-fold) (Table 4SA) and thus downregulation of *comE* in D39ΔStKP may relate with increased production of polysaccharide, a similar correlation with D39ΔPhpP could not be made.

The complete transcription of the *cps* genes depends on the core promoter (CP) immediately upstream *cps2A* and additional elements upstream of CP extending up to the *dexB* gene (51). These other elements constitute (i) the insertional elements (IE), (ii) repeat sequence of pneumococcus (RUP), and (iii) the spacing sequence (SS) (Fig. 7A). Their individual roles vary in the transcriptional regulation of the *cps2A* gene (71). Interestingly, while the deletion of the SS element and core promoter significantly reduces the expression of the *cps2A* gene and the capsule formation, the deletion of the RUP region increases the expression of the Cps2A gene (71). Based on the location of the RUP and the homology between IE and RUP, it is presumed that the RUP may influence gene expression and contribute to the biology of pneumococcus (71). The RUP region thus may participate in such regulation by fine-tuning the transcription of the *cps* locus and CPS production by recruiting a regulatory factor (71), although the precise nature of such a regulatory factor has not been characterized. More recently, several putative regulators, including CcpA, a GntR family transcriptional regulator SPD_0064/cpsR, a MarR family transcriptional regulator SPD_0379, DNA binding protein HU, CodY, GlnR, and RitR, have been identified with the potential binding site within the promoter region of *cps2* (51). The RUP region contains the “cre” binding site, and based on which, we predicted the role of the StkP-phosphorylated CcpA in the repression of the genes. As described above, VncR protein also binds the 218-bp P*cps*, but only in the presence of serum in a type-specific manner (52), and positively regulates the expression of *cps2* genes. It is unknown whether StkP phosphorylates VncR and modulates the VncR-mediated *cps2* gene expression.

Unlike Stk1-mediated phosphorylation of S. aureus CcpA at Thr 18 and Thr 33 (67), the pneumococcus StkP *in vitro* phosphorylated CcpA at its S19, Thr22, and S238 residues. Irrespective of these differences, the *in vitro* reversible phosphorylation of CcpA by StkP and PhpP, and EMSA of the StkP-phosphorylated CcpA reveal the compromised binding ability of CcpA to the *cps2A* promoter and strongly support our hypothesis providing a novel regulatory mechanism of the pneumococcus capsule formation. In addition to the CcpA-mediated regulation of the capsule via binding to the *cre* region in the RUP segment of the core promoter, a recently identified CpsR (SPD_0064) also binds to the RUP region and suppresses capsule expression (51). The CpsR binding region is 15 bp downstream of the *cre* locus, i.e., −146 to −114 bp relative to the transcription start site (TSS) of the *cps* locus. In the absence of PhpP, i.e., in the background of unchecked phosphorylation activity, the transcript abundance of the *spd_0064/cpsR* is upregulated by ~9 folds (Table S2). Thus, the increased binding of CpsR may result in repression of the *cps* locus and capsule formation. However, it is unknown how StkP-mediated phosphorylation modulates the expression of *cpsR*. A defined biochemical link between CcpA and CpsR binding to the *cps2* promoter is presently unknown. The RUP forms a stem-loop structure (99). It is likely that the recruitment of phosphorylated CcpA at the *cre* site of RUP or the possible phosphorylation of CpsR by StkP may affect the folding of its stem-loop structure and alter the binding of CpsR to RUP and, in turn, may result in modulated transcription of the *cps* locus genes and capsule production.

Given this information, we surmised that the increased and decreased expression levels of the polysaccharide capsules genes and capsule contents in D39ΔStkP and D39ΔPhpP mutants is likely due to the altered and reversible phosphorylation-modulated CcpA binding to the region upstream of the *cps2A* gene within its promoter. However, understanding the direct impact of StkP and PhpP on translational and phosphorylation levels of proteins involved in Cps biosynthesis and their interactions with CcpA and other mediators involved in polysaccharide capsule gene transcription via P*cps* in pneumococcus need further investigation.

StkP/PhpP-mediated posttranslational modifications and their regulated metabolic transport, virulence factors, and the capsule formation required for pneumococcal *in vivo* fitness at the mucosal level directly impact pneumococcal adherence, invasion, and evasion processes. However, the modulation of virulence manifestation is both bacteria and host-dependent (100), and like many other Gram-positive pathogens, the virulence of pneumococcus is also multifactorial (6). Thus, besides the mutants’ ability to multiply and survive within the nutritionally variable and hostile host environments, the nature of the virulence phenotype of pneumococcus mutants also depends on host responses to the modulated bacterial cell surfaces. In this study, we have demonstrated that the capsule-depleted D39ΔPhpP mutant displays the wild-type growth pattern but remains attenuated and does not survive in both mouse infection models. D39ΔStkP, on the other hand, although defective in cell division and unable to divide efficiently, show relatively more virulence compared to D39ΔPhpP. Thus, while cell division defects may be crucial for bacterial survival in the host, the bacterium-specific innate immune responses have an equal role in virulence. To that end, we have demonstrated that division/growth defective D39ΔStkP, D39ΔPhpP, and D39ΔMapZ are phenotypically distinct in terms of their ability to evoke host-innate immune responses. D39ΔPhpP, in comparison to D39-WT, D39ΔMapZ, and D39ΔStkP, is unable to elicit important proinflammatory chemokines (Fig. 9C). Similarly, as revealed by NanoString gene expression analysis, the interaction of D39ΔPhpP with the host cells also increases the expression of specific protective chemokine genes (Fig. 9B, Table S5B). The increased expression of CCL2 and CCL20 prevents pneumococcus-mediated sepsis in humans and mice (101–103). Although IL-6 and IL-8 have a protective role by recruiting neutrophils and clearing pneumococcus burden during lung infection (104, 105), overwhelming responses as in the case of D39-WT and comparable responses in the presence of D39ΔMapZ can also cause imbalance resulting in sepsis. With an increased capsule expression level *per se* or by curtailing the exposure of other bacterial components, the highly encapsulated D39ΔStkP mutant may evade this host defense mechanism by dampening the protective proinflammatory responses allowing bacterial survival through other invasive or evasive events (106). Thus, the role of StkP, apart from morphogenesis, is also in allowing bacteria to induce better surface-exposed protein-mediated protective cytokine responses by limiting polysaccharide capsule formation. The PhpP activity, on the other hand, may increase bacterial virulence by promoting capsule formation and invoking inflammatory responses or evading protective innate immune responses.

This study thus elucidates that while StkP is essential for the cell division process in pneumococcus, its kinase activity-mediated posttranslational modification on CcpA serves as a repressor of capsule gene transcription and subsequent capsule biosynthesis. PhpP, however, seems to act as an essential counter enzyme reversing this repressor activity. StkP and PhpP, thus, play crucial roles in fine-tuning the vital cellular functions involved in pneumococcal pathogenesis and maintaining the homeostasis of capsule formation required for the pneumococcal innate immune evasion, survival, proliferation, and dissemination to distant vital organs to cause severe and often fatal diseases. PhpP and StkP can serve as important targets for developing novel therapeutics against pneumococcal infections.

## MATERIALS AND METHODS

### Ethics statement.

This study was carried out per the guidelines outlined in the “Guide for the Care, and Use of Laboratory Animals” of the National Institutes of Health and the NC3R^S^ recommended ARRIVE (Animal Research: Reporting of *In Vivo* Experiments) guidelines (https://www.nc3rs.org.uk/arrive-guidelines). In addition, all animal experiments, anesthesia procedures, and early removal criteria were observed and performed following the protocol (no. 2011A00000051) approved by the Ohio State University Institutional Animal Care and Use Committee (IACUC).

### Bacterial strains, cell lines, and growth conditions.

The wild-type Streptococcus pneumoniae D39 (type 2) (65), D39-derived mutants, and corresponding *phpP/stkP* complemented strains were grown routinely at 37°C in Todd-Hewitt broth (Diffco) supplemented with 0.5% (wt/vol) yeast extract (THY) with or without antibiotics, in a CO_2_ incubator as described previously (36) for maintenance. D39-derived R6 strain, which lacks the polysaccharide capsule, was used as a capsule-negative control strain (42). For pneumococcus transformation experiments and growth curve determination, bacterial cells were grown in a chemically defined medium containing 0.5% yeast extract (C+Y) (36). The antibiotics streptomycin (150 μg/mL), spectinomycin (200 μg/mL), kanamycin (200 μg/mL), and chloramphenicol (5 μg/mL) were used in various experiments during the generation of the mutant and complemented strains. Human lung adenoma epithelial cell lines A549 (CCL-185, ATCC) were cultured and maintained in DMEM medium supplemented with 10% fetal bovine serum and streptomycin-penicillin under the 5% CO_2_ environment using a CO_2_ incubator as described previously (36, 42, 64).

### Generation of recombinant pneumococcal wild-type StkP, PhpP, CcpA, and variant CcpA proteins.

Recombinant StkP and PhpP were obtained as described previously (36). Recombinant His-tag CcpA was produced by cloning the PCR-amplified *ccpA* gene between NdeI and BamHI sites within the multiple cloning sites of the pET14b plasmid (see Table S6 for primers). Four clones of pET14b plasmids containing the entire synthetic wild-type *ccpA* genes and their variant counterparts *ccpAS19A*, *ccpAT22A*, and *ccpAS238A* were custom synthesized (GeneScript, Piscataway, NJ). The plasmids were then used to transform E. coli DH5α and BL21(DE3-PlysS) strain. Recombinant wild-type and variant CcpA proteins were individually expressed. The His-tag-proteins were then affinity purified using Ni-NTA (Nickel-nitrilotriacetic acid) agarose (Qiagen) as described previously (36, 64).

### Generation of PhpP and StkP knockout mutants and complementation.

The markerless *phpP*-knockout mutant, previously derived from an encapsulated S. pneumoniae type 2 strain, D39 (36), was used in the present study. Briefly, the markerless pneumococcus mutant lacking PhpP was created using the Janus-cassette that contained the kanamycin (*kan*) resistance gene, followed by the recessive *rpsL* gene (Sm^S^), and by employing a two-step negative selection strategy (36, 107). In the first step, the Sm^S^-Kan^R^ phenotype of a D39ΔPhpP-Janus strain containing Janus-cassette was obtained after transformation of the laboratory-generated D39-Sm^R^ strain with a DNA construct containing the Janus cassette flanked on either side by an upstream and a downstream region of the *phpP* gene (36). This strain was then subjected to the second step of negative selection, and the Janus cassette was replaced with a PCR product containing the upstream and downstream regions of the *phpP* gene spliced together by spliced-overlap extension (SOE). The resulting Δ*phpP* mutant (phenotype Kan^S^/Sm^R^) was selected on streptomycin-containing blood agar plates. The genetic integrity of the D39ΔPhpP mutant was confirmed as described previously by PCR and DNA sequencing using appropriate screening primer pairs (36).

Unlike the markerless D39ΔPhpP mutant, the isogenic mutant lacking StkP was created by the allelic replacement method by using the pFW6 vector, which has the spectinomycin resistance gene (*aad9*) flanked by two multiple cloning sites (MCS)-I and II and lacks a transcription terminator after the stop codon of *aad9* (108). Briefly, the PCR-amplified 503-bp upstream sequence of *stkP* (primer pairs, StkP-up-F and StkP-up-R) was cloned between the SalI and restriction sites within MCS-I. Similarly, the PCR-amplified 500-bp downstream sequence (primer pairs StkP-Dwn-F and StkP-Dwn-R) was cloned at PstI restriction sites within MCS-II. The PCR-amplified product was obtained using a primer pair, StkP-up-F and StkP-Dwn-R and template pFW(stkP) to transform the competent D39-WT cells to replace the *stkP* gene with *aad9* as described previously (36). The transformants were selected on TSA-blood agar plates containing 200 μg/mL spectinomycin. The genetic integrity of D39ΔStkP mutants was confirmed by PCR, and DNA sequencing using appropriate primers and custom-genome sequencing (BGI Americas). In addition, the absence of the expression of PhpP and StkP proteins was confirmed by Western immunoblotting using the affinity-purified rabbit anti-StkP and anti-PhpP antibodies (36).

To restore the PhpP and StkP expression in the mutants, the genetically confirmed mutants, D39ΔPhpP and D39ΔStkP, were respectively complemented with pDC123 plasmids containing either the wild-type *phpP* or *stkP* gene along with its native RBS (109). Briefly, the *phpP* gene (741 bp), along with its 18 bp RBS, was cloned between KpnI and *Eco*RI sites of the pDC123 vector, using a specific primer pair (pDC-phpP-F and pDC-phpP-R). Similarly, the wild-type *stkP* gene was cloned between the same restriction sites using the primers pairs (pDC-StkP-F and pDC-StkP-R) and cloned into the pDC123 (Chl^R^) plasmid. PhpP and StkP were then expressed under the control of the *cat* gene promoter. The mutant strains (D39ΔPhpP and D39ΔStkP) were made competent in the presence of CPS-1 peptide, transformed with their respective pDC123-*phpP and* pDC123-*stkP* plasmids.

The resultant *phpP/stkP*-complemented mutant strains (D39ΔPhpP::*phpP/*D39ΔStkP::*stkP*) were then selected on blood agar plates containing chloramphenicol (5 μg/mL). The absence of PhpP and StkP proteins in the D39ΔPhpP and D39ΔStkP mutants and their restoration in the complemented strains were confirmed by Western immunoblot analysis and qRT-PCR.

### Generation of MapZ knockout mutant from the D39 S. pneumoniae strain.

D39ΔMapZ mutant was created using a 3,133-bp synthetic gene construct (GenScript, NJ) encompassing upstream and downstream regions of the *mapZ* gene (*spd_0342*) linked in-between with a promoterless *kanR* gene cassette containing its own RBS (ribosomal-binding site). The synthetic gene, therefore, included in tandem (i) the 847-bp upstream region of the *mapZ* gene (*spd_0342*) (i.e., the last 787 residues of the upstream region of *spd_0341*, 12 residues of the intergenic region, and first 48 residues of the *mapZ* gene), followed by (ii) 1,443 residues of the promoterless kanamycin gene (*kanR*) along with its ribosomal binding site (RBS-GGAGGTAAATAA) derived from the pFW13 vector (108), and (iii) the 843-bp downstream region of the *mapZ* gene (i.e., the last 42 residues of *mapZ*, followed by 75 residues of the intergenic region and first 726 bp residues of the downstream gene, *gndA* [*spd_0343*]) (Fig. S2A). This synthetic DNA was cloned between BamHI and SphI sites of the pUC57 plasmid (DmapZ-pUC57). A 3152 bp PCR-amplified product obtained with a primer pair DmapZ-up-F/DmapZ-Dn-R (Table S6) was used to transform the D39 wild-type strain. Colonies of D39ΔMapZ mutant were selected on kanamycin (200 μg/mL)-containing blood agar plates. The genetic integrity of the mutant was confirmed by PCR and sequencing using appropriate flanking primers. (Table S6).

### Generation of double mutants.

Using D39ΔMapZ and D39ΔPhpP mutants, three additional double mutants were derived. To create the D39ΔMapZ-ΔStkP mutant strain, D39ΔMapZ mutant was transformed with the PCR product used for creating D39ΔStkP mutant wherein *stkP* was replaced with the *aad9*. To create D39ΔMapZ-ΔPhpP, D39ΔPhpP was transformed with the PCR product used to create D39ΔMapZ wherein *mapZ* was replaced with *kanR*. Similarly, D39ΔPhpP was transformed using a PCR product with an upstream flanking region of the *phpP* gene and downstream region of the *stkP* gene amplified from D39ΔStkP mutant to obtain D39ΔStkP-ΔPhpP (see Table S6). The double knockout mutants were selected on blood agar plates containing kanamycin and/or spectinomycin described above. D39ΔMapZ-ΔStkP mutant was selected on kanamycin-spectinomycin plates. D39ΔMapZ-ΔPhpP and D39ΔStkP-ΔPhpP mutants were selected on kanamycin, and spectinomycin blood agar plates, respectively. The genetic integrity of these mutants was confirmed by PCR using appropriate primers (Table S6).

### RNA extraction.

For RNA-Seq and quantitative real-time PCR analyses, total RNA was extracted from three independent pneumococcus cultures grown in THY broth at 37°C grown at a mid-log-phase in the CO_2_ incubator. Briefly, the bacterial cultures were pelleted, washed, and adjusted to OD_620_ of 0.6. Next, 10 mL of these normalized cultures were pelleted and resuspended in 200 μL of the lysing buffer (PBS containing 25 μg/mL pneumo-phage lysin) (110) and further incubated for 1 h at 37 C for total lysis. The total RNA was extracted using the Norgen total RNA-extraction kit (Norgen Biotek Corp., Thorold, ON, Canada) per the manufacturer’s instructions. The DNA-free total RNA was obtained by treatment with the RNase-free DNase (Millipore). Qualitative and quantitative analyses of the total RNA were determined by the Agilent RNA6000 bioanalyzer (Agilent Technology, Santa Clara, CA). High-quality total RNA was determined based on RNA integrity (RIN) >7.

### RNA-Seq-based transcriptome analysis.

The DNase-treated total RNA samples were subjected to RNA-Seq analysis in the commercial facility of BGI Americas (Boston, MA, USA). High-quality total RNA (RNA integrity [RIN] >7, 23S/16S >1.0) was determined by the Agilent RNA6000 bioanalyzer (Agilent Technologies), and subsequently, rRNA was removed from each preparation and subjected to corresponding cDNA preparation. Short fragments were made via heat treatment, purified, and resolved using EB (elution buffer) buffer for end repair and the addition of single nucleotide A (adenine). Short fragments were then connected to adapters. Following agarose gel electrophoresis, the suitable fragments were selected for PCR amplification. After confirming the quality and quantity of the sample library, the libraries were sequenced using an Illumina HiSeq 2000 with 100 bp sequencing. Three biological repeats each of D39-WT, D39ΔPhpP, and D39ΔStkP were subjected to RNA-seq transcriptome analysis using the high-quality total RNA (20 μg) preparations,

The primary sequencing data (raw as well as filtered clean reads) produced by the Illumina HiSeq 2000 were essentially analyzed as recently described (64) and aligned with the reference sequence of S. pneumoniae D39 using the SOAP aligner/SOAP2 v2.20 software (62). The alignment of data were used to calculate the distribution of the reads on the reference genes and perform coverage analysis and quantification analysis of gene expression. In addition, Gene ontology enrichment analysis was used for pathway enrichment analysis. The published algorithm assessed the gene expression profile's significance (111). Threshold values for the *P* value and false detection rate (FDR, adjusted *P* value, or q value) < 0.01 and the absolute value Log_2_ ratio ≥1 were used as the cutoff criteria to designate significantly differentiated genes (66). The RNA-seq-based nine transcriptome data files (three each corresponding to the D39-WT, D39ΔStkP, and D39ΔPhpP strains) submitted to the GEO database entry were approved as GSE113337.

Differentially expressed genes (DEG) from the Streptococcus *pneumonia* D39, either ΔStkP or ΔPhpP mutant versus WT were filtered based on fold changes ([up- or downregulated] log2 ±1, and *P* values and corrected *P* values [FDR/q values/adjusted *P* values] < 0.05), and were analyzed via pathway analysis with ClueGO (112, 113) version 2.5.8 in Cytoscape (114, 115) version 3.8.2. The following *S. pneumonia* D39 ontologies were referenced in the pathway analysis: GO_MolecularFunction-GOA_10.05.2017, GO_CellularComponent-GOA_10-05-2017, and GO Biological Process-GOA_10-05-2017. Only pathways with Bonferroni step-down-corrected enrichment/depletion (two-sided hypergeometric test with mid-*P* values less than or equal to 0.05) were displayed.

### Quantitative real-time reverse transcriptase-PCR.

For the quantitative real-time reverse transcriptase-PCR (qRT-PCR) analysis, total RNA was extracted from three independently grown cultures of the wild-type D39 strain and the corresponding isogenic D39ΔPhpP and D39ΔStkP mutants as described above. The first-strand cDNA and subsequent determination of mRNA expression levels of genes were carried out using SYBR green qRT-PCR master mix (Roche), specific primers (Table S6), and a LightCycler 480 (Roche) real-time PCR instrument, as described previously (64). The absence of any PCR-amplified band other than the one expected in a PCR using the D39 genomic DNA as a template confirmed the integrity of primers. The copy numbers of all the genes were normalized to the housekeeping gene, *16S* rRNA. The linear fold change in the mRNA expression levels was analyzed using Exor4 software (Roche), and ≥2-fold decreased/increased transcript abundance was considered a significant change.

### Electron microscopy.

Transmission and scanning electron microscopy (TEM and SEM) of the wild-type, mutant, and *stkP/phpP*-complemented pneumococcal strains were carried out as described previously (64). Briefly, pneumococcus strains were grown to their mid-log-phase, and the bacterial pellets obtained after centrifugation were washed 3 times with 0.1 M cacodylate buffer, pH 7.4, and resuspended in freshly made 0.1 M cacodylate buffer containing 2.5% glutaraldehyde and 4% paraformaldehyde for 30 min on ice followed 4°C for overnight fixation. After fixation, the samples were processed for TEM as well as SEM at the Ohio State University Central Microscope Imaging-Core Facility (CMIF). TEM was performed using a cryo-capable digital TE microscope (Technai G2 Spirit; EFI), and SEM using a field emission gun-equipped SE microscope (Nova SEM400, EFI), as described previously (64). Quantitative analysis of the different morphotypes (lengths and widths) in each strain was conducted manually based on multiple fields of SEM. Similarly, the number of septa per bacterium was counted based on multiple TEM fields. Statistical analysis was conducted based on data obtained from ~20 to 50 SEM/TEM fields, each representing an average length, width, and number of septa from 4 to 6 bacteria by a nonparametric *t* test with Welch’s correction using GraphPad Prism 6.0 software. A *P* value less than 0.05 was treated as a significant difference.

### Immunofluorescence microscopy.

Immunofluorescence microscopy of D39 wild-type, D39ΔStkP, D39ΔPhpP, and corresponding complemented strains was performed to detect the expression level of the type-2 polysaccharide capsule using rabbit type-2 polysaccharide antibody (Statens Serum Institute, Copenhagen). The R6 pneumococcal strain grown to an OD_600_ of 0.5 to 0.6 in 10 mL of THY broth was heat-killed by incubating the culture at 65°C for 2 h, followed by cooling at room temperature (RT). Heat-killed bacteria were then washed, pelleted by centrifugation, and suspended in 1 mL of PBS. The latter was mixed with an equal volume of pneumococcus type-2 rabbit antibodies, incubated overnight at 4°C under constant rotation, and centrifuged. The supernatant containing nonadsorbed antibody specific to type-2 polysaccharide (1:50 dilution) was incubated with the pneumococcal wild-type, mutant, and complemented strains for 2 h, followed by incubation with Cy3-conjugated antirabbit IgG (1:500 dilution) antibody for 1 h to detect the presence of the capsule. The bacterial pellets were washed and stained with 4′,6-diamidino-2-phenylindole (DAPI) dye. The bacterial suspension was then thinly spread on the polylysine-glass slides and air-dried. The stained slides were then observed under a fluorescence microscope (Nikon Eclipse E600) interfaced with a Nikon CCD camera (DSFi1c). Images of the same field were separately captured using red and blue filters and merged using NIS-element (version 4.13) image analysis software.

### Growth curves in the presence of different carbon sources.

Initially, the growth curve patterns of the D39 wild-type, D39ΔStkP, D39ΔPhpP, and *stkP* and *phpP*-complemented mutant strains were determined using a chemically defined C+Y glucose medium (36). Subsequently, D39-WT, and D39ΔStkP, D39ΔPhpP, D39ΔMapZ, D39ΔMapZ-ΔStkP, D39ΔMapZ-ΔPhpP, and D39ΔStkP-ΔPhpP mutant strains were examined in the C+Y medium supplemented with different carbon sources (1% [wt/vol] glucose, maltose, lactose, galactose, and maltodextrin). The fresh culture grown in the chemically defined C+Y medium to mid-log-phase and adjusted to an OD_620_ of 0.6 was diluted to 1:100 in the C+Y medium with different carbon sources. Individual wells of sterile 96-well round-bottom microtiter plates were seeded with 200 μL of the D39 wild-type and various mutant strains diluted cultures. The spectrophotometer was programmed (FluorStar Galaxy software, BMG) to analyze the growth kinetics of culture in each well of the microtiter plate every hour for 15 h at 37°C with an adjustment of horizontal shaking for 10 s before every OD_620_ reading. Growth curves were obtained from three independently grown cultures, each grown in triplicate wells. The initial background values in different groups obtained at the time 0 were subtracted from values obtained at other time points in each group. The corrected values from three individual cultures were used to plot the growth curves using GraphPad Prism-6.

### Extraction of capsule.

The capsular polysaccharide was extracted from late log phase-grown strains of S. pneumoniae. The cultures were pelleted, washed once with PBS by centrifugation, and adjusted to an OD_620_ of 1.0. Each of these cultures was then centrifuged, and the resulting pellet was resuspended in 1 mL of lysis buffer (PBS with 0.2% sodium deoxycholate, 25 μg/mL pneumolysin, 5 μg/mL DNase, 10 μg/mL RNase, and 10 mM MgCl_2_) for 1 h at 37°C. The culture lysates were centrifuged for 15 min at 17,000 × *g* at 4°C. The capsule material was extracted from the supernatant with chloroform (1:1 vol/vol). The upper aqueous phase containing capsule material was removed after centrifugation (17,000 × *g* for 10 min at 4°C) and adjusted to the original starting volume.

### Estimation of capsular contents.

The presence or absence of capsules extracted from the wild-type, single and double mutants and/or complemented pneumococcal strains in whole-cell lysate was qualitatively measured by Western immunoblot analysis. In addition, the presence of polysaccharide capsules was visualized using the capsule type 2-specific antibody described above for immunofluorescence microscopy and corresponding conjugate antibody by the chemiluminescent or chromogenic method, as described previously (64).

The quantitative analysis of the extracted polysaccharide was carried out by measuring the rhamnose sugar content in the extracted capsule as described previously (116). Briefly, 1 mL of diluted samples was mixed with 4.5 mL of 15.86 M sulfuric acid and incubated for 20 min at room temperature. The reaction mixture was then boiled for 10 min and cooled on ice for 20 min. Subsequently, 100 μL of 3% cysteine was added and incubated for 2 h at room temperature. The development of the colored furfural precipitates was spectrophotometrically measured at 430 nm. PBS-containing wells were treated as controls. The rhamnose content in the samples was determined based on the standard curve obtained using different rhamnose concentrations (10 to 100 μg/mL). Results received from three independent experiments, each with three technical replicates, were statistically analyzed by the nonparametric Student's *t* test with Welch correction using GraphPad Prism 6. A *P* value less than 0.05 was treated as a significant change.

### *In vitro* phosphorylation.

*In vitro* autophosphorylation assays were performed using 2 μg of StkP in the presence of 1 μCi γ^32^P-ATP (specific activity 3,000 Ci/mMol, PerkinElmer) at 30°C for 45 min in a final volume of 30 μL phosphorylation buffer (50 mM Tris/HCl, pH 7.5, 1 mM dithiothreitol, 5 mM MgCl_2,_ and/or 5 mM MnCl_2_). The dephosphorylation of StkP was carried out in the presence or absence of an equimolar concentration of PhpP. Similarly, reversible phosphorylation of CcpA was carried out in the presence or absence of StkP, and PhpP, described above. Protein phosphorylation status was determined by SDS-PAGE followed by Coomassie stain and autoradiography.

### *In vivo* phosphorylation of CcpA by StkkP.

*In vivo* phosphorylation of CcpA was performed by expressing the *stkkP* and *ccpA* genes in pCDFDuet-1 vector (Novagen). The *stkkP* gene was cloned between NdeI and KpnI sites within the multiple cloning site-2 (MCS2) using a PCR product obtained with a primer pair, StkkP-F/StkkP-R. The *ccpA* gene was cloned between BamHI and HindIII within the MCS-1 region of the plasmid flanked by 6×His tag-encoding region in the upstream region of the plasmid, using a primer pair, His-CcpA-F/HisCcpA-R. The genetically verified plasmid pCDF-His-CcpA/STKK was used to transform DH5α E. coli and pLysS BL-21 E. coli. CcpA was overexpressed in the BL21 strain under the induction of 1 mM IPTG. The *in vivo* StkkP-phosphorylated CcpA protein was purified by the Ni-NTA affinity column chromatography described above.

Similarly, His-CcpA was expressed in D39ΔPhpP complemented with the wild-type *his*-*ccpA* gene using the pDC123 complementation plasmid (Chl^R^) as described above. *In vivo* phosphorylated His-CcpA was then purified using Ni-NTA chromatography as described above.

### Mass spectrometry analysis.

Purified *in vitro* and *in vivo* phosphorylated His-tagged CcpA proteins bands resolved by SDS-PAGE and Coomassie stained were excised and subjected to in-gel trypsin digestion followed by phosphopeptide enrichment and LC-MS/MS mass spectrometry analysis using the Orbitrap Fusion Tribrid mass spectrometer at the OSU Campus Chemical Instrument Center (CCIC) Mass Spectrometry and Proteomics (MSP) Facility. Briefly, gel pieces were washed in 100 μL of 50% methanol/5% acetic acid for 30 min and then dried and suspended in 75 μL 50 mM ammonium bicarbonate (with 5 mg/mL dithiothreitol [DTT]) for 30 min at RT. The DTT-containing ammonium bicarbonate was aspirated, and the gel pieces were suspended in 50 mM ammonium bicarbonate containing 15 mg/mL iodoacetamide and incubated for 30 min in the dark at RT. The gel pieces were then alternately incubated with 50 mM ammonium bicarbonate and acetonitrile, respectively, for 10 min and then dried. Dried gel slices were rehydrated with 25 μL of 50 mM ammonium bicarbonate and digested with 75 μL of sequencing grade-modified trypsin (10 mg/mL in 50 mM ammonium bicarbonate; Promega, Madison WI) for 6 h at 37°C. The peptides were repeatedly extracted from the polyacrylamide gel with 50% acetonitrile and 5% formic acid. The subsequent phosphopeptide enrichment approach was based on the well-established low pH TiO_2_ enrichment method (117). Enriched phosphopeptides were then subjected to LC/MS-MS analysis using a Thermo-Scientific Orbitrap Fusion mass spectrometer equipped with an EASY-Spray Sources was operated in the positive ion mode. Peptides were separated using mobile phase A (0.1% formic acid in water) and mobile phase B (acetonitrile with 0.1% formic acid) with a flow rate set at 300 nl/min. Typically, mobile phase B was increased from 2% to 35% in 30 min and then increased from 35 to 55% in 5 min and again from 55% to 90% in 5 min and then kept at 90% for another 2 min before being brought back quickly to 2% in 1 min. MS/MS ion scan spectra recorded between *m/z* 400 and 1,600 were generated for the most abundant peaks to determine the amino acid sequence. The full scan was performed at FT mode, and the resolution was set at 120,000 to achieve high mass accuracy MS determination. Sequence information from the MS/MS data were processed by converting the raw files into a merged file (.mgf) using an in-house program, RAW2MZXML_n_MGF_batch (merge.pl, a Perl script). The resulting mgf files were searched using Mascot Daemon by Matrix Science version 2.5.1 (Boston, MA), and the database searched against the most recent Swiss-Prot or NCBI databases (NC_008533.2/CP000410.2, dated 22 Feb 2022). The mass accuracy of the precursor ions was set to 10 ppm. The fragment mass tolerance was set to 0.5 Da. Considered variable modifications were oxidation (Met), deamidation (Asn and Gln) and carbamidomethylation (Cys) and phosphorylation (Ser/Thr/Tyr). Proteins identified with at least 2 unique peptides were considered for reliable identification. A decoy database was also searched to determine the false discovery rate (FDR), and peptides were filtered according to the FDR. Phosphorylated peptides were manually validated, and proteins with a Mascot score of 50 or higher and a *P* value <0.01 with a minimum of two unique peptides from one protein having a -b or -y ion sequence tag of five residues or better were accepted.

### Electrophoretic mobility shift assays.

Electrophoretic mobility shift assay (EMSA) experiments were performed using a ^32^P end-labeled 285-bp probe encompassing the 250-bp region of the promoter element upstream and 37 bp downstream (–250 to +35 nt) of the *cps2A* gene *spd_0315*. These probes were PCR-amplified using a primer pair, PCps2A-F/PCps2A-R. Briefly, the binding of the different concentrations of purified nonphosphorylated CcpA (1 to 3 μM) with the labeled *cps2A* promoter was carried out in the final 40 μl of reaction mixer containing the labeled probe (20,000 CPM/mL) in EMSA buffer (2 mg/mL poly dI-dC [Sigma], 10 mM Tris pH 7.5, 35 mM KCl, 1 mM EDTA pH 7.5, 1 mM DTT, 6% glycerol and 1 mM MgCl_2_). Subsequently, a constant concentration of nonphosphorylated CcpA protein was incubated with different concentrations of StkkP (0.5 to 3.0 μM), followed by incubation with poly di-dC containing EMSA buffer for 5 min at room temperature. The reaction mixture was further incubated at 37°C for 25 min after adding the probe. The CcpA-bound and free probes in the reaction mixtures were resolved by 4.5% nondenaturing polyacrylamide gel electrophoresis (200V × 30 min) using 0.5× Tris-borate-EDTA (TBE) buffer and visualized by autoradiography. In addition, the 100× cold probe was mixed with the labeled probe in the EMSA reaction buffer to determine the specificity of CcpA binding to the P*cps2A* promoter.

### *In vivo* bacterial virulence assay.

The virulence potential of the D39 wild-type and corresponding mutants (D39ΔPhpP and D39ΔStkP) and *phpP/stkP*-complemented strains (D39ΔPhpP::*phpP* and D39ΔStkP::*stkP*) was assessed by employing two mouse infection models. For the septicemia infection model, 17 CD-1 mice (5 weeks old, 20 to 22 g, Charles River Laboratories) were anesthetized with isoflurane and injected with 100 μL of the diluted culture in sterile PBS (5 × 10^3^ CFU) via the retroorbital route. The ability of these pneumococcal strains to cause systemic infection followed by colonization of the nasopharynx was determined by employing the intranasal infection model. As described above, a group of briefly anesthetized 17 mice was infected intranasally with pneumococcal strains (5 × 10^7^ CFU/20 μL). A group of 8 mice that received PBS alone (retroorbitally or intranasally) served as a sham-infected control group. Intranasal infection experiments were performed in two instances (10 and 7 mice per group and housed as 3 to 5 mice/cage). At the end of the experiments, the survival/mortality data were combined for individual mutants and corresponding complemented strains.

The morbidity/mortality for all these groups was monitored 3 to 4 times daily in the first 3 days postinfection and twice daily for 10 days. Experimental animals showing signs meeting the criteria for early removal were euthanized by using compressed CO_2_ gas (CO_2_ pressure 10 to 30% of the chamber volume/min) followed by cervical dislocation to confirm death. The Log-Rank test was applied to statistically analyze the percent survival for each group, and the survival curve was plotted using GraphPad Prism 6 software. The systemic bacterial burden in these animals was determined by counting CFU in an aliquot of 5 μL of blood collected at 24 h and 48h postinfection. Median CFU values obtained from each group (*n* = 10) were statistically analyzed by Mann-Whitney nonparametric unpaired test. The lungs were excised from three animals in each group on day three postinfection (72 h), then fixed in 10% formalin and further processed by the OSU Histopathology Core Facility for tissue sectioning and staining for histopathology. Histopathological slides were examined after Grams and hematoxylin and eosin (H&E) staining to determine the bacterial load and extent of lung pathology. Slides were scanned using Leica ScanScope XT2 high resolution (0.5 μm/pixel) scanner and viewed at ×20 and ×40 magnification using Aperio ScanScope Image viewer.

### NanoString technology-based inflammatory genes expression analysis.

The confluent cultures of A549 cells (CCL-185, ATCC), maintained in 6-well tissue culture plates, were cocultured with D39 wild-type and isogenic mutant D39ΔStkP, D39ΔPhpP, and D39 ΔMapZ pneumococcus strains (multiplicity of infection [MOI] ~100:1 bacteria:cell) for 4 h and total DNase-free RNA was isolated from triplicate tissue-culture samples for each mutant using Norgen total RNA isolation kit as described above. The quantity and quality of RNA samples were assessed by NanoDrop 2000 (Thermo Fisher Scientific, USA). RNA samples were then subjected to multiple inflammatory gene expression analyses using NanoString nCounter inflammation profiling panels (NanoString, USA). Data analysis was carried out with the nSolver Analysis Software 4.0 for the profiling Panel (NanoString, USA) according to the manufacturer’s recommendations. Briefly, samples were examined using 50 ng of total RNA loaded for each sample. Probes for each gene in the panel were allowed to bind and detect the mRNA molecules of that target gene in the sample and copy numbers of mRNA molecules were counted by NanoString technology. The gene-expression data were normalized in two steps; positive-control normalization (with internal positive-control sequences) and housekeeping normalization, differential expression plots, fold change values for each gene, and other analyses were obtained using the nSolver Analysis Software version 4.0 (NanoString, USA). The list of genes used for analysis is given in Table S8 in the supplemental material, with corresponding annotations.

### Meso Scale Discovery analysis of cytokines/chemokines.

The concentrations of IFN-γ, TNF-α, and 8 different interleukins (IL-1-β, IL-2, IL-4, IL-6, IL-8, IL-10, IL-12p70, IL-13) in culture supernatants of A549 cells cocultured with D39-WT and other derived single and double mutants collected at 0, 2, and 4 h time intervals were analyzed using V-plex Plus proinflammation panel 1 (human) kit (C4049-1) (Meso Scale Diagnostic, Rockville, MD) based on an electro chemiluminescent detection method according to the manufacturer’s recommendations. All collected samples were stored at −80°C, thawed, and diluted 1:3 before analysis. Data were collected and analyzed using MSD Meso 1300/SQ 120 Quickplex Microplate Reader equipped with Discovery workbench data analysis software (MSD, Rockville, MD) and located in the OSU CCTS-supported Analytical & Development Laboratory. Briefly, 96-well microtiter plates were coated with linker-coupled capture antibodies (specific to analyte listed above and provided by the manufacturer) for 1 h and then aspirated and washed with washing buffer (PBS/0.05%Tween 20) 3 times. Standards and supernatants (25 μL) were added to appropriate wells, incubated for 1 h with shaking, and washed again 3 times with washing buffer. Detection antibodies were added to each well and incubated for 1 h at room temperature and washed, as described above. Finally, 150 μL of a reading buffer was added to each well. The plate was analyzed on the MDS instrument. Standard curves were formed by fitting electrochemiluminescence signals from calibrators, and analyte concentrations were extrapolated from a standard curve calculated using a four-parameter logistic fit using MSD Workbench 3.0 software. Data were then plotted and statistically analyzed using GraphPad Prism-6 software.

### Data availability.

RNA-seq analysis data are deposited in GEO database (GEO Accession no. GSE113337).
