# Supplementary material for: StkP- and PhpP-Mediated Posttranslational Modifications Modulate the S. pneumoniae Metabolism, Polysaccharide Capsule, and Virulence
Source: Infect Immun. 2023 Mar 6;91(4):e00296-22. doi: 10.1128/iai.00296-22 (PMC10112228; doi:10.1128/iai.00296-22)
Supplement: Supplemental file 6 — Tables S6 and S7 and Fig. S1 to S3. Download iai.00296-22-s0006.pdf, PDF file, 1.5 MB [file iai.00296-22-s0006.pdf]

Table-S6: A. Sequences of the oligonucleotides used for constructing recombinant proteins, and D39 strain-derived mutants

Primer-name	Primer sequence- 5'-3'	Purpose
StkP-up-F	TTT*GTCGAC*ATTGAAAATCAAAAGATT	Upstream region of *stkP* (spd_1542) between salI &XhoI
StkP-up-R	TTT*CTCGAG*TTACATTCTGCATCCTCCTCG	
StkP-Dwn-F	AAA*CTGCAG*AAGCAGATGGATAATCAA	downstream region of *stkP between EcoRI and PstI* (spd_1542)
StkP-Dwn-R	TTTCTGCAGGGAGCACTAAAGGTCGCA	
StkkP-F	AAA*CATATG*ATCCAAATCGGCAAGATT	to generate kinase region specific stkP gene to be inserted between NdeI and KpnI sites of MCS-2 in pCDF-duet-1 vector for In vivo phosphorylation
StkkP-R	TTT*GGTACC*TTAAAATCTATGTTTTTCGGTG	
His-StkkP-F	AAA*CATATG*ATCCAAATCGGCAAGATT	PCR product to be inserted into pET14 B between BamH1 to make His-Tag StkP (31).
His-StkP-R	AAA*GGATCC*TTAAGGAGTAGCTGAAGT	
pDC-phpP-F	ATC*GGTACC*ATCGGATTAGGAAGGAAC	Wild-type *phpP (spd_1543)* between KpnI and EcoRI for complementation
pDC-phpP-R	ACC*GAATTC*TCATTCTGCATCCTCCTC	
pDC-StkP-F	TTT*GGTACC*CCTTGTTTCTATGAACGA	Wild-type *stkP (spd_1542)* between kpnI and EcoRI for complementation
pDC-StkP-R	AAA*GAATTC*TTAAGGAGTAGCTGAAGT	
DmapZ-up-F	AGAAACACTATGCTCGCCCAGAAG	To obtain PCR product containing upstream and downstream regions with truncated (containing *kanR*) or intact *mapZ* (*spd_0542*) to create D39ΔMapZ or D39 ΔMapZ-ΔPhpP using D39ΔPhpP mutant
DmapZ-Dn-R	ACGGCTCAAGATATCAGCTGTGAT	
MapZ-Scrn-F	GAGTTTAAGATTGAGGTCTCT	For screening the integrity of the Δ*mapZ* mutant
MapZ-Scrn-R	TGCTAGCATGTACACGTTC	
MapZ-F	ATGAGTAAAAAAAGACGAAATCGTCAAG	To generate the entire *mapZ* gene
MapZ-R	TTAGTAGTCCAAGTCATCCGCATGA	
CcpA-F	TTT*GGATCC*GATGGATGCAGATGATACAGTA	*ccpA or variant* gene cloning between BamHI and HindIII within the MCS-1 of pcdF-Duet-1 vector or pET14B to generate rec HisTag protein
CcpA-R	AAA*AAGCTT*CTATTTACGTTTTCGTGTTGA	
PCps2A-F	AAAAAGATTATACCACATTGTGTA	Promoter sequence of *cps2A*
Pcps2A-R	CTTTCTGTGAACGTGATTTTT	
pDC-HisCcpA-F	TTT*GGTACC*AGGAGAGATGCATCATCATCAT CATCATCACATGGATGCAGATGAT	Wild-type *ccpA* with its RBS between kpnI and EcoRI
pDC-CcpA-R	ACC*GAATTC*CTATTTACGTTTTCGTGTTGA	
PhpP-up-F	TTTGCATGCTTAAGGAGCAGGGGCATT	Upstream region of phpP (Spd_1543) and downstream region of *stkP* (spd_1542) to create D39ΔStkP-ΔPhpP mutant using D39ΔPhpP mutant
StkP-Dwn-R	TTTCTGCAGGGAGCACTAAAGGTCGCA	
StkP-up-F	TTT*GTCGAC*ATTGAAAATCAAAAGATT	Upstream and downstream region of *stkP* (spd_1542) to create D39 ΔMapZ-ΔStkP using D39ΔMapZ mutant
StkP-Dwn-R	TTTCTGCAGGGAGCACTAAAGGTCGCA	

Table-S7. The sequence of oligonucleotides used for qRT-PCR analysis.

SPD_ORF#	Gene ID	Forward (5′-3′)	Reverse (5′-3′)
1543	*phpP*	AAACAAGGGCAACCGTAATG	TGCTCAATAGTGACGGCTTG
1542	*stkP*	AATGGTTGCAGGAGTTCTGG	TGCCGAAGGATTTCTCGAGT
0315	*cps2A*	TGTCGCTGTTTTAGCAGATA	TAACCGTCAAATCGGTATTC
0319	*cps2E*	CATGCAGGGTGGAATGTTTAAGG	AGCTCGTCCAAGCTAGTCTTC
0328	*cps2L*	GGTGAGGATTTTATTGGTGA	TGGATCCTTCACTTGGTAAC
0301	*regR*	GAATCGATCCCGATGAAAA	AGCCTGTTGTCCTTCCTCAA
0063	*strH*	GGTTTTTCCGTTGGCAGTAA	GCTCAAACGCATCGTAGACA
1046	*lacG2*	GCCCTGCCTACTAAATATCC	CCAAACTACCACCATTGACT
1633	*galT2*	GATCGAATCTACCTGACCAA	GAGTCACCAAGTCCATCAAT
1504	*nanA*	ATTTGCCAATTGAAGGGTTG	CTCCTACCGAAGCGAAAGTG
1797	*ccpA*	GGTCAATGGCAATAAAAATG	CCAAACTCGAAAAATAACCA
House keeping gene	*16S RNA*	CGCGAAGAACCTTACCA	ACCCAACATCTCACGACACG

The open reading frame (ORF) numbers are based on the genome sequence data of D39 *S. pneumoniae* strain.

Figure-S1
Sequence changes observed in the 4th serial passage of D39ΔPhpP mutant.

```
D39ΔPhpP passage-4
Scaffold154_1    Deletion    236   236   NC_008533   1995571    1995572    G     -
          0     0     0     0
          ||||||||||||||||||||||||||||||||||||||||||||||||||-
     ||||||||||||||||||||||||||||||||||||||||||||||||||
199546     TAGATAGTACCATGAACCATTGTTTTGGAGCCATCCTGTCGCCATAGCAC
           TAGATAGTACCATGAACCATTGTTTTG*AGCCATCCTGTCGCCATAGCACC
```

complement(1995045..1997150) spd_2017 CbpA

```
>NC_008533.2:1995045-1997150 Streptococcus pneumoniae D39, complete sequence
TTAGTTTACCCATTCACCATTGGCATTGACTCCATAGCCATCTACAGTTGTGTTGACTGCAAGGGCACCTGAGCCATTGACATA
GTACCATTTATCTGATACTTTGAACCATTGGCTTGCTTTCATAGCACCTGATGCTTCAAGATAGTACCAGGTATCTCCATCTTT
CACCCAACCTGTCGCCATATCACCATTAGCGTTGAGGTAATACCATGAACCGTTGTATTGGAGCCATCCTGTCGCCATATCACC
ATTAGCGTTGAGGTAGTACCATGAACCGTTGTTTTGGAGCCATCCTGTCGCCATATCACCATTAGCGTTGAGGTAGTACCATGA
GCCATTGTATTGGAGCCATCCTGTCGCCATAGCGCCATTGCTGTTTAGGTAGTACCATGAACCATTGTATTGGAGCCATCCTGT
CGCCATAGCACCATTAGCGTTTAGGTAGTACCATGAACCATTGTTTTGGAGCCATCCTGTTGCCATTGAACCATTAGCGTTTAG
ATAGTACCATGAACCATTGTTTTGGAGCCATCCTGTCGCCATAGCACCATTAGCGTTTAGATAGTACCATGAACCGTTGTTTTG
GAGCCATCCTGTTGCCATTGAACCATCAGTATTGTAGAAGTACCACATACCGTTTTCTTGTTTCCAGCCTGTTTTTGGAGTAGA
TGGTTGTGCTGGTTTTTCAGTTTTTGGCGGTTGCTGTTGAGTCAAGCGATTATATTCTTCTTCTGATCTACGAGCATAGTCTTC
TTCAGCTTGTTGATCATCTGTTTTTTCTGCTTTTGGTTGTTCAGCTGGCTTCTCTGGTTTTGGAGCTGGTTTTTCTGGTTGAGT
AGCCGGCGCTGGTTGTGGTTGTTCAGCTGGTTTTTCTTTAACTTTATCTTCTTCTGCTGCTTTTCGTTTAGCTTCTTCTTCTGC
TTTTTTACGATCTGTCTTGATGTTTTCTAACCTTGTAGCCTCAGCTTTTTTACTCTCAACTTTCGCTTTTGCTTGCTTAATTTT
TTCCTCGTCTCGAGGTTCCTTAGCTTCCTCTTTTACTAGTTCAAGCTCCGCTTCTTTAACTTTCACATCGGACTCAGCAATTTC
AAGGTCAAGCGTTTTGTAAGTATTGGTTGGGTAGTTACGGCGATCTTCTTCTTTTTGATCCTTGGCTTTTTTCTCAGCTTCTTC
AACCTTCTTCTCAGCTTCTGCTACCTTTTTTCCTGATTTCAGGGATGAGCTTGGAAGAGTTTCTTCACCTACGCTAGAATCTGA
AGACTTCGCATCATTTTCTTTTTTATCAGGTGTTGCTAGCTCTCCAGGAACTCCTCGTTTTGCCCGCCCCTTTGGTTTACCTTG
ATCTGAAGTCGCTACATTAGCTTCCTTCAACTTAGCATCTGCTTTTCGTTTAGCTTCTTCTTCTGCTTTTTTACGATCTGTCTT
GATGTTTTCTAACCTTGTAGCCTCAGCTTTTTTACTCTCAACTTTCTCTTTTGCTTGCTTAATTGTGCCCTCGTTTCGAGATTC
TTTAGCTTCCTCTTTTACTAGTTCAAGCTCCGCTTCTTTAACTTTCACATCGAACTCAGCAATTTCAAGTTCAAGCGTTTTGTA
AGTATTGGTTGGGTAGTTACGACGATCTTCTTCTTTTTGATCCTCGGCTTTTTTCTTAGCTTCTTCAACCTTCTTCTTAGCTTC
TGCTACCTTTTCTCCTGGTTTCAATGTATCTTTTTTAAACTTCTCAAAAGCTGCGTCTAACTTTGCTTTTATTTCTGACGGCAA
CTCATCTTTCGACTTCTCTTCTAAAACATTTAATTCACGCAAATACTTCGTTTTAATTGCGCTCAACTTTATGTTTAAGGCGAC
ATTTTGGGTATGTTTTCTTCTATCTAGTTGAATCTCCCTCAACATTTTTCTATATATTCATCGACGACTTGTTTAGCAGCTTT
CCTATGTTCTGTCTTTGCCATATTAGAAGAAGTGGCTGCTTGGGTACTTCCCTCGTTCTCTGTCGCATGAACCACACTTCCCAT
AACAAGACTGGCAACAGCTACACTAGCTACTCCAATACTAAATTTACGAATTGAATAATGTACTTTTCTTTCGCTTTTTGATGC
AAACAT
```

D39- wt

```
MFASKSERKVHYSIRKFSIGVASVAVASLVMGSVVHATENEGSTQAATSSNMAKTEHRKA
AKQVVDEYIEKMLREIQLDRRKHTQNVALNIKLSAIKTKYLRELNVLEEKSKDELPSEIK
AKLDAAFEKFKKDTLKPGEKVAEAKKKVEEAKKKAEDQKEEDRRNYPTNTYKTLELEIAE
FDVKVKEAELELVKEEAKESRNEGTIKQAKEKVESKKAEATRLENIKTDRKKAEEEAKRK
ADAKLKEANVATSDQGKPKGRAKRGVPGELATPDKKENDAKSSDSSVGEETLPSSSLKSG
KKVAEAEKKVEEAEKKAKDQKEEDRRNYPTNTYKTLDLEIAESDVKVKEAELELVKEEAK
EPRDEEKIKQAKAKVESKKAEATRLENIKTDRKKAEEEAKRKAAEEDKVKEKPAEQPQPA
PATQPEKPAPKPEKPAEQPKAEKTDDQQAEEDYARRSEEEYNRLTQQQPPKTEKPAQPST
PKTGWKQENGMWYFYNTDGSMATGWLQNNGSWYYLNANGAMATGWLQNNGSWYYLNANGS
MATGWLQNNGSWYYLNANGAMATGWLQYNGSWYYLNSNGAMATGWLQYNGSWYYLNANGD
```

MATGWLQNNGSWYYLNANGDMATGWLQYNGSWYYLNANGDMATGWVKDGDTWYYLEASGA
MKASQWFKVSDKWYYVNGSGALAVNTTVDGYGVNANGEWVN-

D39-phpP

MFASKSERKVHYSIRKFSIGVASVAVASLVMGSVVHATENEGSTQAATSSNMAKTEHRKA
AKQVVDEYIEKMLREIQLDRRKHTQNVALNIKLSAIKTKYLRELNVLEEKSKDELPSEIK
AKLDAAFEKFKKDTLKPGEKVAEAKKKVEEAKKKAEDQKEEDRRNYPTNTYKTLELEIAE
FDVKVKEAELELVKEEAKESRNEGTIKQAKEKVESKKAEATRLENIKTDRKKAEEEAKRK
ADAKLKEANVATSDQGKPKGRAKRGVPGELATPDKKENDAKSSDSSVGEETLPSSSLKSG
KKVAEAEKKVEEAEKKAKDQKEEDRRNYPTNTYKTLDLEIAESDVKVKEAELELVKEEAK
EPRDEEKIKQAKAKVESKKAEATRLENIKTDRKKAEEEAKRKAAEEDKVKEKPAEQPQPA
PATQPEKPAPKPEKPAEQPKAEKTDDQQAEEDYARRSEEEYNRLTQQQPPKTEKPAQPST
PKTGWKQENGMWYFYNTDGSMATGWLQNNGSWYYLNANGAMATGWLQNNGSWYYLNANGS
MATGWLQNNGSWYYLNANGAMATGWLQYNGSWYYLNSNGAMATGWLQYNGSWYYLNANGD
MATGWLKTTVHGTTSTLMVIWRQDGSNTTVHGITSTLMVIWRQVG-

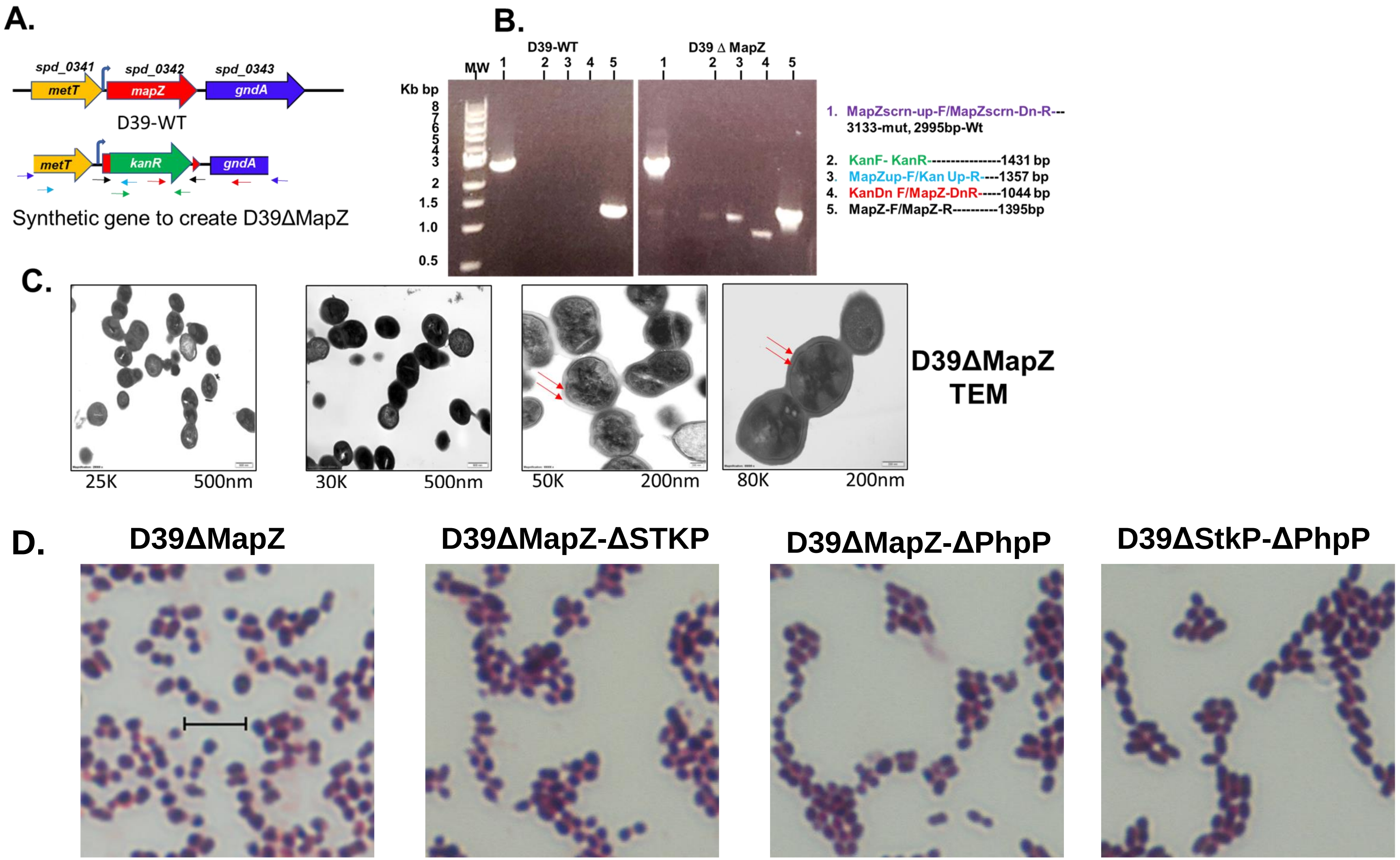

Fig. S2. Creation and Characterization of D39ΔMapZ mutant from encapsulated D39 wild-type *S. pneumoniae*. (A) Based on the D39-WT genome, a synthetic gene construct was designed for the allelic replacement of *mapZ* with *kanR as* detailed in the Materials and Methods section. Color coded arrows indicate the primer pairs designed to determine genetic integrity of the mutant. **(B)** PCR products obtained using primer pairs as indicated were resolved on1% agarose gel with EtBr. **(C)** Transmission electron microscopy (TEM) of D39ΔMapZ mutant observed at different magnifications as indicated. Bar sizes equivalent to 200nm or 500 nm as indicated under each picture. Arrows show the presence of capsule. (**D)** Bright field microscopy of Gram-stained D39 mutant lacking MapZ, MapZ & StkP, MapZ & PhpP, and StkP & PhpP. The mutants were derived as described in Materials and Methods. For all panels Bar=2µm

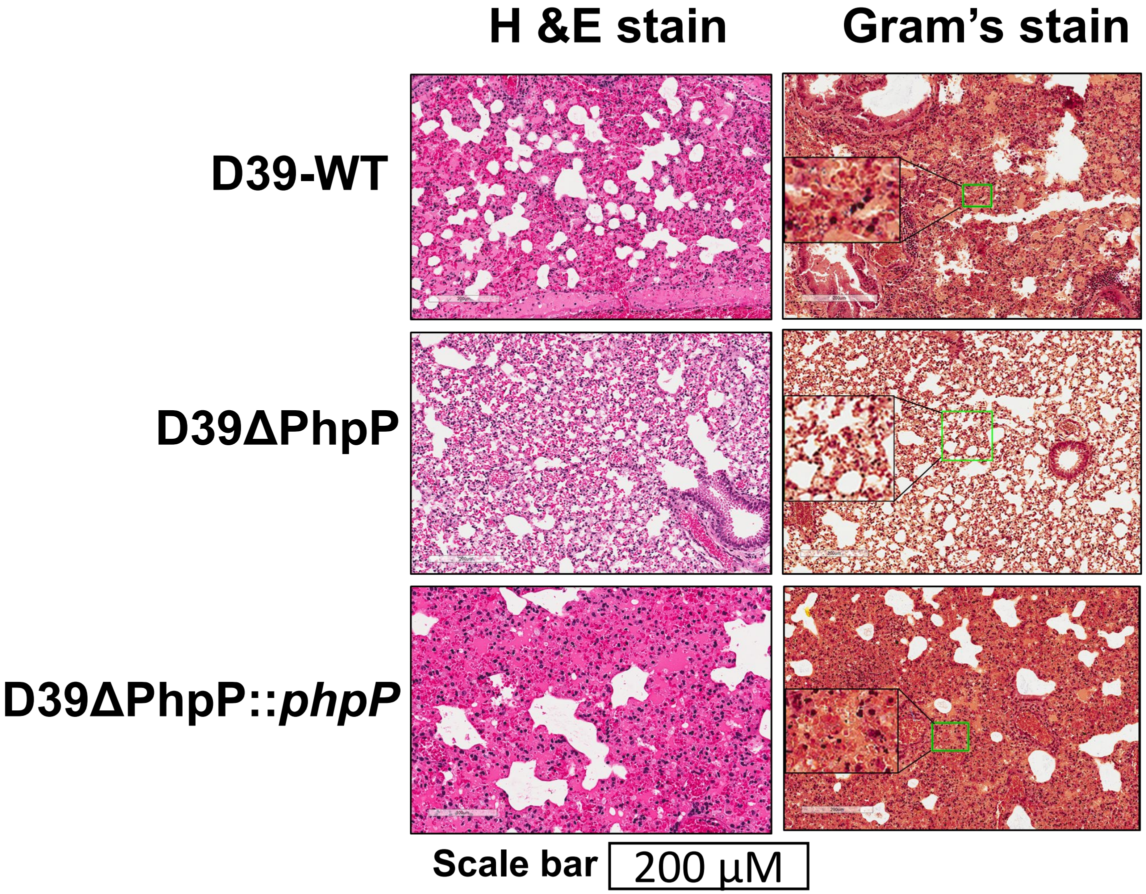

Fig S3. Histopathology of lung tissues obtained from mice retroorbitally infected with pneumococcus strains. Gram-stained, and Haematoxylin and Eosin-stained sections of lung tissues obtained from retroorbitally infected mice with D39-WT, D39ΔPhpP and D39ΔPhpP::*phpP* pneumococcal strains as indicated. (20X magnification, at 0.5µm pixel size). Marked insets of the Gram-stained lung sections highlights the presence (D39–WT, D39ΔPhpP::*phpP*) or absence (D39ΔPhpP) of magnified and visible Gram-positive *S. pneumoniae.*
